# Impact of Processing on Nutritional and Functional Properties of Hemp and Sunflower Oilcakes

**DOI:** 10.3390/foods15213791

**Published:** 2026-10-23

**Authors:** Klausz Ronald Balla, Mădălina Ungureanu-Iuga, Silvia Mironeasa

**Affiliations:** 1 Faculty of Food Engineering, “Ştefan cel Mare” University of Suceava, 13th Universitatii Street, 720229 Suceava, Romania; klausz.balla1@student.usv.ro (K.R.B.); silviam@fia.usv.ro (S.M.); 2 Integrated Center for Research, Development and Innovation in Advanced Materials, Nanotechnologies, and Distributed Systems for Fabrication and Control (MANSiD), “Ştefan cel Mare” University of Suceava, 13th Universitatii Street, 720229 Suceava, Romania; 3 Mountain Economy Center (CE-MONT), “Costin C. Kiriţescu” National Institute of Economic Researches (INCE), Romanian Academy, 050711 Bucharest, Romania

**Keywords:** hemp (*Cannabis sativa* L.), sunflower (*Helianthus annuus* L.), oilcakes, protein valorization, techno-functional properties, extrusion texturization, life cycle assessment, circular bioeconomy

## Abstract

Global environmental challenges and expanding dietary demands have driven interest toward upcycling agri-food by-products into sustainable, functional ingredients within circular bioeconomy frameworks. Oilcakes derived from industrial hemp (*Cannabis sativa* L.) and sunflower (*Helianthus annuus* L.) processing are rich in storage globulins (edestin and helianthinin), balanced polyunsaturated fatty acids, dietary fibers, minerals, and bioactive phenolics. However, their broad adoption in human foods remains constrained by low protein solubility, rigid insoluble fiber structures, antinutritional compounds (such as phytic acid and condensed tannins), off-flavors, and chlorogenic acid-induced oxidative greening. This review comprehensively examines the nutritional, structural, and techno-functional evolution of hemp and sunflower press cakes across primary oil extraction routes (cold pressing, hot expelling, solvent extraction, supercritical, and enzyme-assisted aqueous extraction) and downstream processing operations (thermal treatments, extrusion cooking, solid state/submerged fermentation, and ultrafine micronization). While cold pressing and supercritical extraction preserve native globular folding, solubility, and interfacial activity, thermal expelling and solvent desolventization induce severe protein denaturation and hydrophobic aggregation. Secondary interventions, particularly high moisture extrusion and microbial bioprocessing, facilitate protein texturization into anisotropic fibrous networks, increase soluble fiber ratios, degrade antinutritional complexes, and mitigate beany/bitter sensory attributes. Furthermore, Life Cycle Assessment (LCA) and techno-economic benchmarks underscore the environmental and economic advantages of upcycled oilseed proteins over conventional soy and dairy isolates. Strategic integration of green extraction, computational modeling, and formulation science offers a viable pathway for valorizing crude oilcakes into clean label, high-value functional ingredients for novel plant-based food and food colloids.

## 1. Introduction

The growing global population and escalating environmental pressures have intensified the demand for sustainable, plant-based protein sources and high-value nutritional ingredients [1,2]. Traditional oilseed processing primarily focuses on the extraction of vegetable oils, leaving behind vast quantities of protein-rich solid residues known as oilcakes or press meals [3,4]. Industrial hemp (*Cannabis sativa* L.) and sunflower (*Helianthus annuus* L.) represent two high-yielding, agriculturally resilient crops whose by-products hold exceptional potential for valorization within circular bioeconomy models [5,6]. Historically utilized as low-value animal feed or discarded as agricultural waste, hemp and sunflower oilcakes are rich in high-quality storage proteins, essential fatty acids, dietary fiber, minerals, and bioactive phenolic compounds [7,8].

However, raw oilseed press cakes present techno-functional and sensory limitations that hinder their direct incorporation into human food systems [9,10]. These include poor protein solubility, undesirable flavor profiles, high fiber content, and the presence of antinutritional factors such as phytates and polyphenolic complexes [11,12]. The nutritional value, structural behavior, and functional dynamics—such as water and oil holding capacities, emulsification, foaming, and gelation are heavily dictated by the primary oil extraction methods (e.g., cold pressing, hot pressing, solvent extraction, supercritical CO_2_) and subsequent processing technologies (e.g., thermal treatment, extrusion, fermentation, micronization) applied to the biomass [13,14].

This comprehensive review evaluates the structural, nutritional, and techno-functional evolution of hemp and sunflower oilcakes across various primary extraction and downstream processing pathways. By examining the biochemical mechanisms underlying processing-induced modifications, this paper elucidates how physical, chemical, and enzymatic treatments can be tailored to upcycle these agricultural by-products into high-value functional ingredients for novel plant-based foods, bakery goods, and sustainable food matrices.

Industrial hemp (*Cannabis sativa* L.) and sunflower (*Helianthus annuus* L.) represent two high-yielding, agriculturally resilient crops whose by-products hold exceptional potential for valorization within circular bioeconomy models [5,6]. These two oilseeds were specifically selected for this review because they provide non-major allergen alternatives to conventional soybean proteins, with complementary amino acid profiles rich in arginine and sulfur-containing amino acids [15,16]. Moreover, they embody two distinct and critical techno-functional bottlenecks in plant protein science: sunflower proteins are severely restricted by chlorogenic acid-mediated oxidative discoloration and covalent cross-linking [17,18], while hemp proteins are challenged by dense hull lignocellulose and oxidative volatile off-flavors [19,20].

To identify relevant literature, comprehensive bibliographic searches were conducted across Scopus, Web of Science, PubMed, and Google Scholar spanning publications from 2005 to 2026. Primary search strings combined Boolean operators with targeted keywords, including: (‘hemp’ or *‘Cannabis sativa’* or ‘sunflower’ or *‘Helianthus annuus’*) and (‘oilcake’ or ‘press cake’ or ‘press meal’ or ‘by-product’) and (‘extraction’ or ‘extrusion’ or ‘fermentation’ or ‘thermal processing’ or ‘functional properties’ or ‘techno-functional’). Peer-reviewed research articles and critical reviews addressing the compositional profiles, extraction technologies, downstream structural/functional modifications, and circular food system applications of hemp and sunflower oilcakes were prioritized. Studies focusing exclusively on agronomic traits, non-food biofuel/feed applications, or psychoactive cannabinoid pharmacology were excluded.

The final bibliographic search was performed in July 2026. Around 180 articles were studied from 4 databases; from those entries, we kept 163 after eliminating duplicates and those deemed unfit for this article. The final set of papers included in the review was selected through a two-stage screening process: titles and abstracts were independently screened for relevance, followed by a full-text evaluation against the predefined inclusion and exclusion criteria. This manuscript is intended as a comprehensive narrative review.

## 2. Composition and Nutritional Characteristics of Hemp and Sunflower Seeds

### 2.1. Hemp Seed Characteristics

Hemp seeds (*Cannabis sativa* L.) possess a distinct nutritional profile characterized by high concentrations of protein (20–25%), lipids (25–35%), and dietary fiber (20–30%) [21,22]. The primary lipid fraction is rich in polyunsaturated fatty acids (PUFAs), notably linoleic acid (18:2 n-6) and *α*-linolenic acid (18:3 n-3), maintaining an optimal n-6 to n-3 ratio between 2:1 and 3:1, which is considered ideal for human cardiovascular health [23,24]. The storage proteins of hemp seed consist predominantly of edestin, a hexameric legumin-type globulin accounting for approximately 60–80% of total protein, and albumin (20–30%) [25,26].

From an amino acid perspective, hemp protein contains all essential amino acids required for human nutrition, exhibiting high levels of glutamic acid, arginine, and aspartic acid, alongside sulfur-containing amino acids (methionine and cysteine) that are often limiting in other leguminous plant proteins [16,27]. The dehulled hemp seed matrix offers superior protein digestibility due to the removal of the fibrous outer hull, whereas whole hemp seeds contain substantial insoluble fiber fractions, primarily cellulose and lignin [19,28].

### 2.2. Sunflower Seed Characteristics

Sunflower seeds (*Helianthus annuus* L.) represent a major global oilseed crop, valued for their lipid content (40–50%) and high-quality protein fraction (20–25%) [15,29]. The lipid profile is dominated by unsaturated fatty acids, primarily oleic acid and linoleic acid, with specific high-oleic cultivars exhibiting exceptional thermal stability due to elevated monounsaturated fatty acid proportions [30,31].

The protein composition of sunflower seed is dominated by helianthinin, an 11S globulin representing roughly 50–70% of total soluble protein, alongside a 2S albumin fraction comprising 10–30% [17]. Helianthinin possesses a globular hexameric quaternary structure stabilized by non-covalent interactions and disulfide bonds [32]. Sunflower protein displays a favorable essential amino acid pattern, rich in sulfur-containing amino acids and tryptophan, though it is traditionally limiting in lysine [15,32]. The seed hull contains elevated levels of insoluble fiber, lignified structures, and wax compounds, which are often removed during dehulling to improve protein extractability and meal quality [33].

### 2.3. Bioactive Compounds and Antinutritional Factors

#### 2.3.1. Phenolic Profiles and Bioactive Distribution

Hemp and sunflower seeds contain diverse arrays of non-nutrient bioactive secondary metabolites that exhibit antioxidant, anti-inflammatory, and radical scavenging properties, while simultaneously acting as techno-functional modulators in processed food matrices [24,28]. Hemp seeds are characterized by a distinct polyphenolic profile dominated by phenylpropionamides (notably cannabisin derivatives such as cannabisin A, B, C, and D), *N*-*trans*-caffeoyldopamine, and flavonoids including quercetin, luteolin, and apigenin glycosides [28,34]. These structures possess multiple phenolic hydroxyl groups capable of scavenging reactive oxygen species (ROS) and donating electrons to stabilize lipid radicals [35,36]. In contrast, the soluble polyphenol fraction of sunflower seeds is dominated by phenolic acids, where chlorogenic acid (5-*O*-caffeoylquinic acid; CGA) accounts for around 77% of the total polyphenolic mass, accompanied by smaller quantities of caffeic, ferulic, and quinic acids [37,38,39].

Beyond these primary soluble phenolics, a significant portion of the bioactive compounds in both hemp and sunflower matrices exists in bound forms, covalently esterified to cell wall structural polysaccharides or entrapped within the lignin complex [34,35]. Industrial oil extraction and subsequent downstream processing significantly impact the distribution, stability, and bioaccessibility of these non-nutrient metabolites [24,40]. While exhaustive solvent extraction generally concentrates total phenolics within the defatted meal, excessive thermal exposure during desolventization or roasting can trigger the degradation and decarboxylation of sensitive phenolic acids, thereby reducing the overall radical scavenging capacity of the resulting press cake [41,42]. Conversely, controlled processing interventions such as extrusion cooking, enzymatic hydrolysis, and solid-state microbial fermentation can actively cleave these bound ester and glycosidic linkages [11,43]. This structural liberation not only increases the concentration of free, extractable antioxidant compounds but also enhances their metabolic bioaccessibility within the gastrointestinal tract, transforming the crude press meal into a high-value source of dietary phytonutrients for functional food formulations [11,34].

#### 2.3.2. Molecular Mechanisms of Chlorogenic Acid Oxidation and Protein Cross-Linking

**Figure 1:**
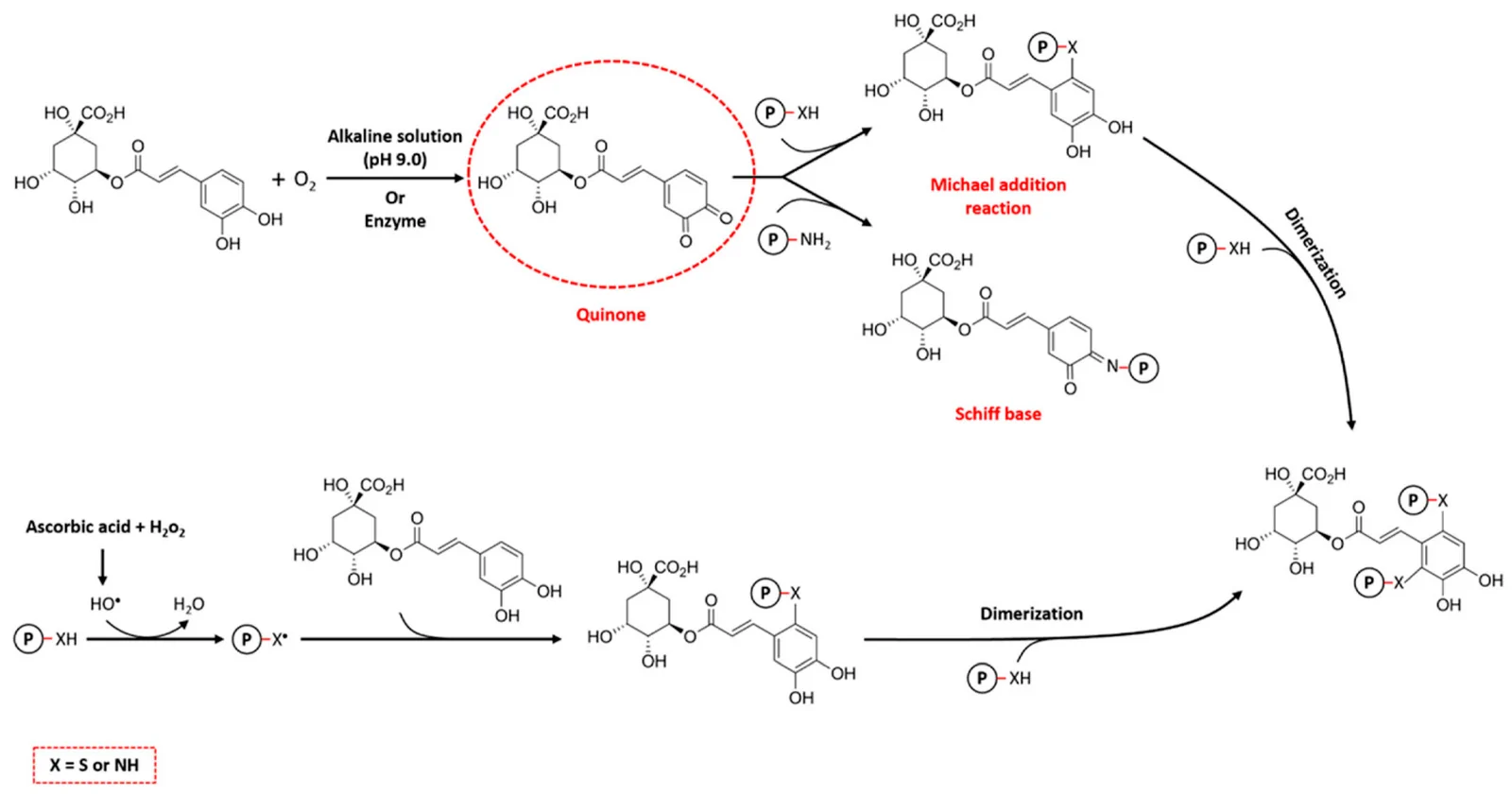
The mechanisms of covalent reactions between proteins and chlorogenic acids (CGAs) [46]. Reproduced from Tarahi et al. [46], under the terms of the Creative Commons Attribution 4.0 International (CC BY 4.0) license.

The technological utilization of sunflower seed proteins is heavily restricted by the chemical reactivity of chlorogenic acid (CGA) during alkaline aqueous extraction and processing [17,18]. Under neutral to alkaline processing regimes (pH > 7.0) in the presence of molecular oxygen, CGA undergoes rapid enzymatic or non-enzymatic autoxidation: the ortho-diphenolic moiety (catechol group) of the caffeoyl residue is oxidized into a highly electrophilic ortho-quinone intermediate [12,38]. These reactive *o*-quinones subsequently participate in irreversible nucleophilic addition (Michael-type addition) reactions with free nucleophilic amino acid side chains on sunflower storage proteins, principally the *ε*-amino groups of lysine residues, the imidazole rings of histidine, and the sulfhydryl (−SH) groups of cysteine residues within the helianthinin (11S) globulin and 2S albumin fractions [29,44]. This covalent binding pathway induces conformational shifts and extensive steric cross-linking, producing a characteristic dark green trihydroxyindole-type pigment complex, which darkens to brown upon further oxidative polymerization [15,18]. At the molecular level, covalent attachment of bulky, oxidized polyphenolic adducts modifies the net isoelectric point (pI) and surface electrostatic charge (ζ-potential) of the protein by consuming positively charged basic residues (Lys^+^), leading to altered repulsive forces, premature precipitation, and severe reductions in aqueous protein solubility [12,44]. In addition to covalent pathways, non-covalent interactions driven by hydrophobic stacking between the aromatic rings of CGA and non-polar amino acid residues (shown in Figure 1) (leucine, isoleucine, phenylalanine), as well as multi-point hydrogen bonding between phenolic hydroxyls and protein carbonyl/amide backbones, further promote macromolecular aggregation, mask active cleavage sites for digestive proteases, and reduce in vitro gastrointestinal protein digestibility [17,45].

#### 2.3.3. Phytate–Mineral Chelation and Protease Inhibition

Antinutritional factors present in hemp and sunflower matrices include phytic acid (*myo*-inositol 1,2,3,4,5,6-hexakisphosphate; IP_6_), condensed tannins, and endogenous enzyme inhibitors, which collectively impair micronutrient absorption and macronutrient utilization [7,11]. Phytic acid is the primary storage form of phosphorus in oilseeds, localized predominantly inside the protein storage vacuoles (aleurone grains) of the cotyledon cells [47]. Structurally, the IP_6_ molecule features six phosphate ester groups with twelve ionizable protons exhibiting widely distributed acid dissociation constants (pK_a_ values from <2 to ~9.5), which confers an exceptionally dense negative electrostatic surface charge across the entire physiological gastrointestinal pH range [11,21,47]. This high polyanionic charge density drives powerful chelation reactions with multivalent mineral cations, preferentially coordinating with divalent metals in the stability order:

Zn^2+^ > Cu^2+^ > Ni^2+^ > Co^2+^ > Mn^2+^ > Fe^2+^ > Ca^2+^ > Mg^2+^

At neutral-to-basic luminal pH values in the small intestine, phytic acid readily forms insoluble, planar, multi-dentate organometallic coordination complexes with zinc (Zn^2+^), iron (Fe^2+^/Fe^3+^), and calcium (Ca^2+^), rendering these essential micronutrients bio-inaccessible for enterocyte transporter uptake [19,21].

Beyond direct mineral sequestration, phytic acid interacts electrostatically with seed storage proteins: at pH values below the protein isoelectric point (pH < pI), negatively charged phosphate groups bind directly to positively charged amino acid side chains (Arg^+^, Lys^+^, His^+^), whereas at pH < pI, ternary protein–cation–phytate bridges form via divalent mineral intermediates (Ca^2+^, Mg^2+^) [27,48]. These ionic interactions stabilize the globular core of edestin and helianthinin against thermal unfolding, restrict substrate accessibility within catalytic clefts, and directly inhibit digestive enzymes (pepsin, α-amylase, and trypsin) by chelating essential catalytic cofactors (Ca^2+^ for trypsin stability) or by electrostatically complexing with the proteases themselves [11,49].

## 3. The Influence of Extraction Methods on Oilcake Quality

### 3.1. Main Oil Extraction Methods

**Cold pressing** involves mechanical expression using screw presses or hydraulic expellers maintained at low processing temperatures (typically <50 °C) without chemical solvents [50,51]. This physical method preserves the native tertiary and quaternary structures of oilseed proteins, minimizes thermal oxidation of residual lipids, and avoids chemical contamination [52]. However, cold mechanical pressing exhibits limited oil recovery efficiency, leaving substantial residual fat contents (more than 10%) within the resulting cake [51,53].

**Hot pressing** incorporates thermal pre-treatment steps, such as cooking, roasting, or conditioning prior to mechanical expelling, elevating process temperatures in the range of 80–100 °C [40,54]. Thermal conditioning reduces oil viscosity and ruptures plant cell walls, yielding higher oil recovery rates compared to cold pressing [52]. Nevertheless, elevated thermal exposure induces extensive protein denaturation, hydrophobic aggregation, lipid oxidation, and Maillard browning reactions within the press meal [40].

**Solvent extraction** utilizes organic non-polar solvents, traditionally n-hexane or emerging bio-based alternatives like ethanol and ethyl acetate, to solubilize and recover oil from pre-pressed meals [55,56]. Achieving near-complete lipid extraction (<1% residual fat), this technique produces highly defatted meals [57]. However, industrial solvent desolventization and thermal toaster stages often trigger structural denaturation of delicate globulin fractions [55,58].

Although contact with non-polar solvents (e.g., n-hexane) at ambient temperatures causes limited direct conformational disruption, the subsequent industrial desolventization-toasting stage, which employs direct steam and temperatures exceeding 105–110 °C to recover the solvent, triggers extensive thermal denaturation [55,58,59]. This harsh thermal history alters secondary and tertiary structures, causing loss of ordered *α*-helices, exposure of buried non-polar residues, and formation of intermolecular *β*-sheet aggregates stabilized by covalent disulfide cross-linking [36,49]. Consequently, this treatment depresses the nitrogen solubility index (NSI 43.1%) and interfacial activity, although the evacuation of lipids creates a porous capillary network that enhances Oil Holding Capacity (OHC > 1.18–1.34 g/g) [35,60,61].

**Supercritical fluid extraction** (SF-CO_2_) employs carbon dioxide above its critical pressure (7.38 MPa) and temperature (31.1 °C), operating as an environmentally benign, non-toxic, and tenable non-polar solvent [62,63,64]. SF-CO_2_ facilitates near-complete oil removal (<2% residual fat) at mild operating temperatures, preventing thermal damage to proteins, protecting lipophilic bioactives, and yielding solvent-free oilcakes with clean functional profiles [65,66].

**Enzyme-assisted aqueous extraction** (EAAE) utilizes cell-wall-degrading enzymes (cellulases, pectinases, hemicellulases) or proteases in aqueous media to disrupt the seed wall matrix and hydrolyze lipid-protein complexes [67,68]. EAAE operates under mild thermal and pH conditions, enabling concurrent oil isolation and the recovery of highly soluble, partially hydrolyzed protein fractions without toxic chemical inputs [67].

### 3.2. Influence of Extraction Methods on Oilcake Properties

#### 3.2.1. Protein Content and Quality

The primary oil separation mechanism dictates both the concentration and conformational integrity of the residual protein fraction [3,69]. Intensive defatting pathways such as non-polar solvent extraction and SF-CO_2_ extraction concentrate the crude protein content of hemp and sunflower defatted meals up to 50.2–55% on a dry weight basis by exhaustively removing the lipid phase [69,70,71]. In contrast, cold mechanical pressing yields lower protein concentrations (24.8–36.1% dry matter) due to higher residual oil entrapment [51,53].

From a macromolecular perspective, the thermal history of the extraction method governs conformational stability. Fourier-transform infrared (FTIR) spectroscopy deconvolution of the Amide I vibrational band (1600–1700 cm^−1^) demonstrates that cold pressing and SF-CO_2_ preserve native secondary structures, maintaining a balanced distribution of *α*-helices (~1650–1658 cm^−1^) and intramolecular β-sheets (~1630–1638 cm^−1^) in edestin and helianthinin [69,72]. Conversely, hot pressing and industrial solvent desolventization subject meals to temperatures exceeding 80–100 °C , triggering denaturation characterized by the loss of ordered *α*-helices and the emergence of strong bands associated with intermolecular *β*-sheet aggregates (~1618–1628 cm^−1^ and ~1685 cm^−1^) [36,49]. Furthermore, intrinsic tryptophan fluorescence exhibits a distinct bathochromic (red) shift in maximum emission wavelength (λ_maX_) from ~330 nm to 345 nm, confirming that compact hydrophobic cores unfold and expose aromatic fluorophores to the polar solvent, leading to covalent disulfide (S-S) cross-linking and reduced essential amino acid availability [72,73].

#### 3.2.2. Residual Oil Content and Fatty Acid Composition

Residual lipid concentrations range widely depending on the mechanical and thermodynamic efficiency of the extraction system: cold pressing leaves 8–15% residual fat, hot pressing yields 5–10%, whereas hexane and SF-CO_2_ leave <2% [50,63]. The fatty acid profile of cold-pressed cakes remains rich in polyunsaturated fatty acids (PUFAs), predominantly linoleic (C18:2 n-6) and α-linolenic (C18:3 n-3) acids in hemp, and oleic (C18:1) and linoleic acids in sunflower, preserving their natural bioactive and nutritional value [24,31].

However, high residual fat contents in cold- and hot-pressed press meals accelerate susceptibility to oxidative degradation during post-processing storage [74]. High-temperature mechanical pressing and desolventization promote the homolytic cleavage of allylic C-H bonds, accelerating the formation of lipid alkyl radicals that react with molecular oxygen to generate primary oxidation intermediates, measured as elevated conjugated dienes and peroxide values (PV) [75,76]. Over time, these unstable lipid hydroperoxides decompose into volatile secondary oxidation products (hexanal, 2,4-heptadienal, and octen-3-one) that impart rancid odors and can covalently interact with protein amino groups via lipid–protein co-oxidation pathways [75,77].

#### 3.2.3. Dietary Fiber Composition

The carbohydrate and dietary fiber distribution of the resulting cake depends on mechanical dehulling efficiency and whether chemical or enzymatic reagents are used during extraction [78]. Conventional mechanical pressing and non-polar solvent extractions leave the structural cell wall matrix intact, resulting in a cake dominated by insoluble dietary fiber (80–90% IDF of total fiber), primarily cellulose, cross-linked xyloglucans, and complex lignified polyaromatics [19,78]. In contrast, enzyme-assisted aqueous extraction (EAAE) actively remodels this carbohydrate architecture [67].

The inclusion of multi-component cellulases, pectinases, and hemicellulases during aqueous extraction cleaves internal *β*-(1 → 4) and *α*-(1 → 4) glycosidic linkages within the plant cell wall, partially hydrolyzing high-molecular-weight structural polysaccharides into shorter-chain soluble dietary fibers (SDF) [79,80]. This enzymatic shift substantially raises the SDF:IDF ratio, improving the water-binding capacity, swelling index, and physiological fermentability of the recovered cake by gut microflora [78,81].

#### 3.2.4. Mineral and Micronutrients

Mineral fractions (K, Mg, Ca, P, Fe and Zn) naturally concentrate within defatted oilseed cakes following lipid removal, making the press meal an abundant micronutrient reservoir [19,82]. Mechanical pressing and SF-CO_2_ extraction preserve native mineral chelates and lipid-soluble micronutrients (such as α- and γ-tocopherols and phytosterols) without leaching or oxidative degradation [51,66].

In contrast, aqueous extraction and wet fractionation processes can lead to the loss of water-soluble minerals (K^+^, Na^+^) and B-complex vitamins into aqueous discharge streams [68]. However, where phytic acid co-concentrates with minerals in the protein fraction, strong electrostatic binding sequesters divalent cations (Zn^2+^, Fe^2+^ and Ca^2+^) into insoluble organometallic complexes [21]. Processing routes that incorporate mild hydrothermal conditioning or endogenous phytase activation help hydrolyze the inositol phosphate ring, liberating these bound micronutrients and improving their in vitro bioaccessibility [11].

#### 3.2.5. Bioactive Compounds and Antioxidant Activity

The retention and antioxidant efficacy of polyphenolic compounds such as cannabisin lignanamides in hemp and chlorogenic, caffeic, and ferulic acids in sunflower are directly dictated by the extraction thermal regime and solvent polarity [35,38]. Cold pressing and SF-CO_2_ operate under mild thermal conditions that preserve thermolabile monophenols and flavonoids, maintaining high total phenolic content (TPC) and strong radical-scavenging capacities (DPPH, ABTS, and FRAP assays) in the residual cake [42,66].

Hot pressing and thermal toasting cause the thermal degradation and decarboxylation of sensitive phenolic acids, while simultaneously promoting the release of bound phenolics from insoluble cell wall matrices [40,42]. In sunflower cake extraction, alkaline aqueous processing triggers the rapid oxidation of chlorogenic acid into electrophilic *o*-quinones, which react covalently with nucleophilic protein residues (*ε*-amino groups of lysine), reducing the concentration of free, active antioxidant compounds in the soluble phase [18,44].

#### 3.2.6. Protein Solubility

Protein solubility, quantified across the pH spectrum as the nitrogen solubility index (NSI), serves as an essential functional metric governing emulsification, foaming, and gelation performance [16,45]. Protein isolates derived from cakes produced via cold pressing and mild SF-CO_2_ maintain classic U-shaped solubility profiles with high NSI values at both acidic (pH 2.0–3.0; >60%) and alkaline (pH 8.0–10.0; >80%) ranges, with minimal solubility around the isoelectric point (pH 4.0–5.0) [45,69].

High-temperature hot pressing and commercial hexane desolventization drastically depress the entire NSI curve, reducing solubility across all pH values to <20–30% [69,72]. Thermal denaturation disrupts the hydration layer surrounding globular proteins, exposing internal non-polar residues (Leu, Ile, Phe) to the solvent [83]. This leads to extensive hydrophobic aggregation and covalent intermolecular disulfide (S-S) cross-linking, which stabilizes insoluble macrogels that resist re-solubilization in aqueous media [39,84].

#### 3.2.7. Water and Oil Holding Capacity

Water Holding Capacity (WHC) and Oil Holding Capacity (OHC) reflect the balance between exposed hydrophilic groups, surface non-polar patches, and internal capillary structures within the cake [13,35]. Native cold-pressed meals exhibit moderate WHC (1.5–2.5 gH_2_)/g dry matter) because polar amino acid residues and hydrophilic hydroxyls on cellulose are packed inside compact protein globules and rigid cell walls [51,85]. Controlled thermal processing during hot pressing partially unfolds these protein globules, exposing buried peptide backbones and polar side chains (−COOH, −NH_2_, −OH), which increases direct hydrogen bonding with water and elevates WHC to >3.0–4.5 g/g [13,83].

OHC is governed by the exposure of non-polar amino acid residues and matrix porosity [86]. In cold-pressed meals, residual lipids (8.9–16.4%) fill internal structural pores, limiting the physical capacity to bind additional fat [51,53]. Exhaustive defatting via solvent extraction or SF-CO_2_ evacuates these porous structural micro-cavities while exposing non-polar amino acid side chains, increasing OHC to >2.5–4.0 g oil/g dry matter [35,60,87]. This high oil-binding performance makes thoroughly defatted oilcake isolates functional for fat retention in formulated meat analogs and bakery systems [88,89].

#### 3.2.8. Emulsifying and Foaming Properties

The interfacial activity of protein isolates and concentrates derived from oilcake proteins, measured by Emulsifying Activity Index (EAI), Emulsion Stability Index (ESI), Foaming Capacity (FC), and Foam Stability (FS), relies on molecular flexibility and rapid diffusion to oil–water and air–water boundaries [90]. Cold-pressed and SF-CO_2_-derived proteins maintain flexible globular conformations, enabling them to migrate rapidly to the interface, reorient, and form a continuous, viscoelastic protective film that resists droplet coalescence and Ostwald ripening [77,91].

Conversely, severe thermal exposure during hot pressing promotes the formation of large, rigid protein aggregates with high molecular weights [83]. These aggregates diffuse slowly toward interfaces and are unable to unfold effectively at oil–water or air–water boundaries, resulting in lower EAI values, broader emulsion droplet size distributions (d_43_ > 10 μm), and rapid foam collapse [13,92]. Furthermore, residual lipids present in cold-pressed cakes act as foam destabilizers by competitively displacing proteins from the air–water interface without providing dilatational elasticity, rendering defatted protein concentrates more suitable for high foam aeration applications [51,85].

#### 3.2.9. Rheological and Textural Properties

The rheological behavior of oilcake aqueous dispersions expressed through steady-state shear viscosity (*η*), dynamic storage modulus (*G′*), and loss modulus (*G″*) is governed by protein solubility, particle size distribution, and fiber swelling [18,93]. Native protein isolates extracted from cold-pressed cakes exhibit shear-thinning (pseudoplastic) flow behavior (n < 1.0) and, upon thermal curing (>75–85 °C), undergo ordered gelation where *G′* exceeds *G″* by over an order of magnitude, producing elastic, cohesive three-dimensional hydrogels [18,94,95].

Thermally damaged meals derived from hot pressing and solvent desolventization contain pre-denatured, aggregated proteins that cannot form coherent cross-linked gel networks upon heating [83]. Instead, they produce weak, turbid, particulate suspensions with elevated initial apparent viscosities and high yield stresses, but low gel elasticity and high susceptibility to syneresis [81,94]. In contrast, enzyme-assisted aqueous extraction produces partially hydrolyzed peptide fractions that reduce dispersion viscosity, enabling higher solids loading in liquid nutritional beverage applications without excessive thickening [67,96].

When analyzing the textural behavior of matrices enriched with hemp oilseed cake derivatives, precise mechanical evaluations demonstrate significant alterations in hardness, cohesiveness, and compression characteristics [7]. For example, texture profile analyses (TPA) of food systems incorporating hemp oilseed components indicate a distinct rise in structural firmness due to high dietary fiber and coarse particle fractions. Specifically, the inclusion of hemp derivatives increases product hardness values while lowering cohesiveness and altering compression resilience, as larger plant particles disrupt continuous protein–starch matrices [97].

Beyond fluid rheological behavior, the physical texture of oilcakes ranging from compact, rigid solid expeller pellets to crumbly, semi-solid defatted meals is strongly dictated by residual oil, moisture content, and processing history [51]. Cold-pressed expeller cakes retain residual lipids (8–15%) that act as internal plasticizers, yielding softer, more cohesive, and malleable structures that deform under compressive force [51]. In contrast, hot-pressed and solvent-extracted meals undergo intense defatting and thermal desolventization, resulting in dense, rigid, and brittle cakes characterized by elevated hardness and fracturability, which require greater mechanical energy for downstream milling and pulverization [55,98]. When reconstituted into thermally induced hydrogels, native protein isolates derived from cold pressing undergo ordered unfolding to form elastic, firm, and cohesive three-dimensional gel networks with high structural integrity [94,95]. Conversely, severely pre-denatured press cakes undergo disordered particulate agglomeration, resulting in weak, crumbly gels with negligible firmness and high susceptibility to syneresis [81,94,95].

#### 3.2.10. Color Changes

Extraction parameters directly govern the chromatic attributes (L^*^,a^*^,b^*^ coordinates) of oilseed press cakes [19]. Cold mechanical pressing and SF-CO_2_ preserve natural seed pigments, producing light green-tan flours from dehulled hemp seeds and pale cream-gray flours from dehulled sunflower kernels [19]. High-temperature hot pressing and solvent desolventization accelerate non-enzymatic Maillard browning reactions between reducing sugars and protein amino groups, reducing lightness (L^*^) and increasing red-brown chromaticity (+a^*^, +b^*^) [40].

In sunflower cakes, color modification is driven by the autoxidation of chlorogenic acid (CGA) [38]. Under neutral to alkaline extraction conditions (pH > 7.0), CGA oxidizes into ortho-quinones, which react with helianthinin amino side chains to form intense green trihydroxyindole pigments that turn dark brown upon drying [18,44]. Preventing this discoloration requires extraction under acidic conditions (pH < 5.5) or pre-extraction washing with aqueous ethanol to yield light colored, visually acceptable food ingredients [15,18].

#### 3.2.11. Flavor and Aroma

The volatile flavor profile of oilseed press cakes is shaped by residual lipid oxidation and thermal processing reactions [20,77]. Cold-pressed hemp and sunflower cakes retain volatile terpenes and alkyl alcohols that impart mild, nutty, and slightly grassy aroma notes characteristic of raw seeds [19,20]. Hot pressing and thermal toasting generate volatile pyrazines, pyrroles, and furans via Maillard pathways, producing desirable roasted, toasted aromatic profiles [40].

However, during prolonged storage or high-temperature processing in the presence of air, residual polyunsaturated fatty acids undergo oxidation, yielding secondary volatile aldehydes (hexanal, (*E,E*)-2,4-decadienal), ketones, and short-chain alcohols that generate bitter, rancid, and paint-like off-notes [33,75]. Hexane-extracted meals can also retain solvent residues if desolventization is incomplete, whereas SF-CO_2_ extraction eliminates residual solvent contamination and helps strip non-polar volatile off-flavors, producing deodorized protein flours with neutral taste profiles [63,77].

From a regulatory and food safety perspective, hexane extraction requires stringent desolventization to comply with strict Maximum Residue Limits (MRLs) established for human foodstuffs (e.g., ≤1–10 mg/kg in defatted flours under EU Directive 2009/32/EC and US FDA guidelines) [55]. Growing consumer demand for solvent-free ingredients and the occupational hazards of volatile organic compounds have stimulated shifts toward greener bio-solvents (ethanol, ethyl acetate) and supercritical fluids [55,62]. Nutritionally, while hexane strips over 98% of neutral lipids, the small remaining lipid fraction is enriched in polar phospholipids and free fatty acids that are prone to accelerated thermal oxidation during desolventization, elevating primary oxidation markers (conjugated dienes and hydroperoxides) [75,76]. Moreover, the desolventizing heat promotes advanced non-enzymatic Maillard cascades between reducing sugars and reactive *ε*-amino groups of lysine, reducing essential amino acid bioavailability and generating dark melanoidin pigments [40,49].

#### 3.2.12. Storage Stability

The storage stability and shelf life kinetics of oilseed cakes are dictated by residual moisture content, water activity (a_w_), and residual lipid levels [99,100]. Cold-pressed cakes containing 8–15% residual oil are prone to hydrolytic rancidity catalyzed by residual seed lipases and non-enzymatic autoxidation of polyunsaturated fatty acids, which increases free fatty acid (FFA) values, peroxide values (PV), and thiobarbituric acid reactive substances (TBARS) during ambient storage [19,74].

Exhaustively defatted meals (<2% fat) produced via solvent extraction or SF-CO_2_ exhibit superior oxidative stability during long-term storage [99]. To prevent protein aging, structural cross-linking, and microbial growth, cake moisture content must be maintained below 8–10% (a_w_ < 0.60) within hermetic, oxygen-barrier packaging [99,100]. Controlling storage temperature and relative humidity preserves high protein solubility and functional performance over extended industrial shelf life periods [19,100].

### 3.3. Comparative Analysis of Extraction Methods

In modern commercial setups, the standard industrial pathway differs significantly between the two oilseeds [52,55]. For commodity sunflower processing, the global standard is a two-stage pre-press solvent extraction scheme: high-throughput mechanical screw pressing extracts 60–70% of the oil, followed by continuous counter-current n-hexane extraction to deplete residual cake lipids below 1.5% [52,55,101]. In contrast, commercial hemp processing operates predominantly via single-stage mechanical screw cold pressing (<50 °C), driven by consumer demand for unrefined, cold-pressed virgin oils and non-denatured protein-rich press meals [50,51]. Supercritical CO_2_ and enzyme-assisted aqueous routes are currently confined to specialized, high-value nutraceutical markets due to high capital expenditure and enzyme costs [62,67].

The selection of an optimal primary extraction process involves balancing nutritional retention, functional performance, operational cost, and environmental sustainability.

A comparative analysis regarding the impact of the primary extraction methods on nutritional composition, protein functionality and sustainability profiles of oilseed meals is shown in Table 1.

## 4. Processing Technologies Applied to Oilcakes

Downstream processing technologies are frequently deployed to modify, refine, and functionalize hemp and sunflower oilcakes, converting raw, crude press meals into high-performing, tailored food ingredients [9,14]. In their unprocessed state, crude press cakes present significant techno-functional and organoleptic limitations, including poor nitrogen solubility, high levels of rigid insoluble dietary fiber, bitter off-flavors, and antinutritional complexes that restrict their direct incorporation into human food matrices [11,12,33]. To overcome these structural barriers, secondary processing interventions such as controlled thermal conditioning, solid-state and submerged microbial fermentation, high-temperature short-time extrusion cooking, and ultrafine mechanical micronization are employed to induce targeted molecular reorganizations [41,43,102]. These unit operations systematically disrupt the compact native quaternary assemblies of core storage globulins (edestin and helianthinin), cleave complex lignocellulosic networks, enhance the ratio of soluble to insoluble dietary fiber, and degrade heat-labile antinutrients [72,80]. Consequently, the strategic application of these processing platforms allows manufacturers to precisely modulate surface hydrophobicity, charge density, interfacial film formation, and viscoelastic flow, thereby expanding the utility of oilcake-derived concentrates and isolates in complex food colloids, high volume bakery goods, and texturized plant-based meat analogs [103,104,105].

**Figure 2:**
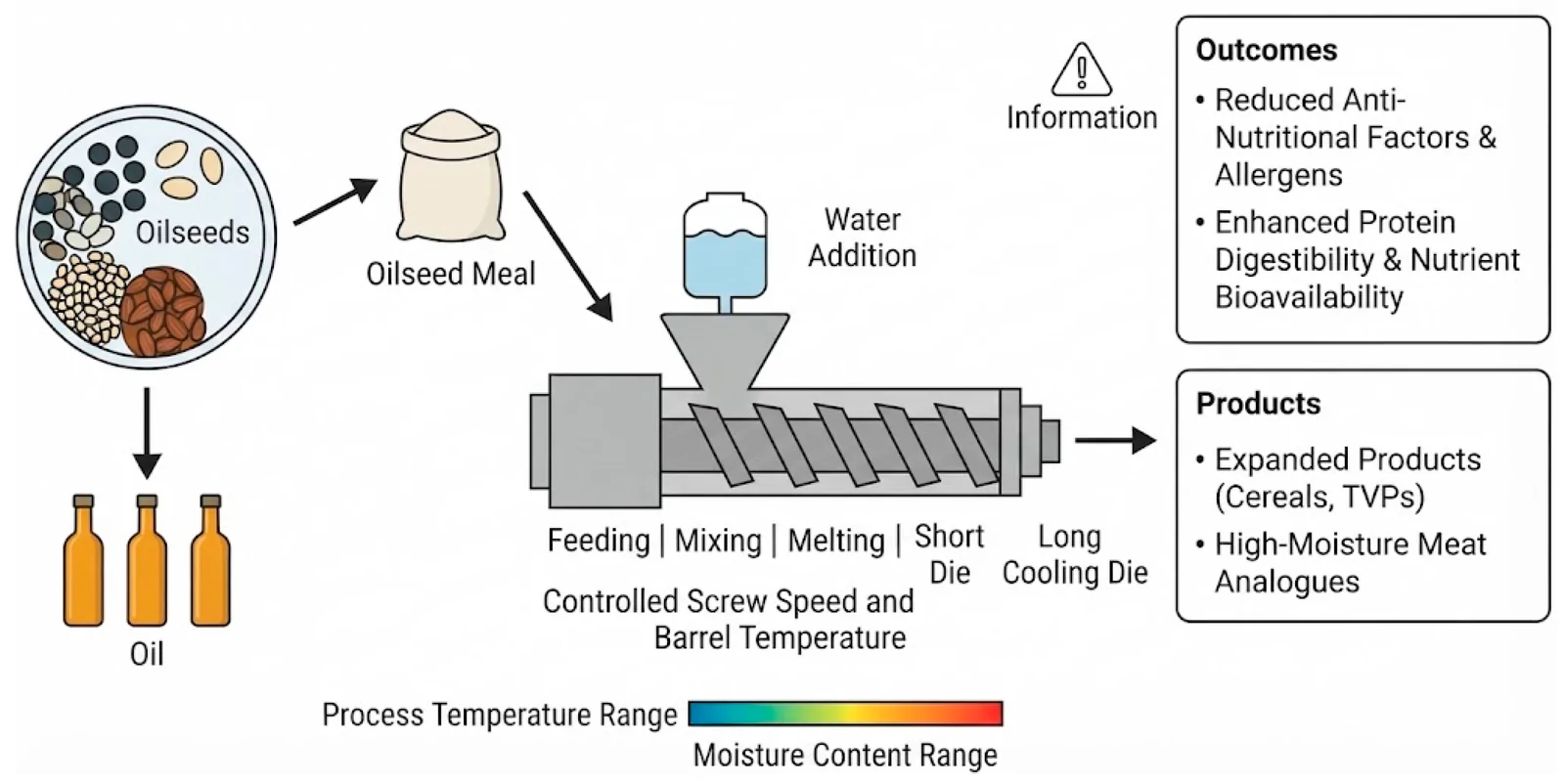
Process flow and structural modifications during extrusion cooking of hemp and sunflower press meals. Adapted from Kadam et al. [14].

The process flow and structural modifications during extrusion cooking of hemp and sunflower press meals are shown in Figure 2.

### 4.1. Primary Processing Technologies

#### 4.1.1. Thermal Processing

Thermal processing encompassing conventional oven roasting, dry heating, autoclaving, and radio frequency treatments serves as a primary technique for modifying the techno-functional properties of Phaseolus vulgaris, which could be applied also to hemp and sunflower press cakes [40,83]. Exposure to elevated temperatures induces the thermal denaturation of globular storage proteins (edestin and helianthinin), destabilizing their quaternary and tertiary structures through the disruption of non-covalent interactions such as hydrogen bonds and hydrophobic associations [36,106]. As polypeptide chains unfold, previously buried internal hydrophobic amino acid residues and free sulfhydryl groups are exposed to the aqueous microenvironment, prompting intermolecular disulfide exchange and the formation of high-molecular-weight aggregates [49,73].

The operational mode of thermal delivery plays a decisive role in directing structural outcomes. While moist heat (e.g., autoclaving, steam conditioning) facilitates plasticization, controlled conformational rearrangement, and the destruction of heat-labile antinutritional factors (such as trypsin inhibitors) at relatively lower activation energies, excessive dry roasting can lead to severe protein carbonization and advanced Maillard reactions [36,107]. These non-enzymatic browning cascades between protein free amino groups (*ε*-amino groups of lysine) and reducing carbohydrates generate cross-linked melanoidin complexes, which can darken the meal and reduce essential amino acid bioavailability [40,41].

#### 4.1.2. Fermentation

Biochemical modification via solid-state fermentation (SSF) and submerged fermentation using selected starter cultures such as *Lactiplantibacillus plantarum*, *Aspergillus oryzae*, and *Rhizopus oligosporus* represents an eco-friendly bioprocessing route to enhance the nutritional and functional profile of oilcakes [108,109]. During microbial metabolism, secreted endogenous extracellular proteases, phytases, and carbohydrases hydrolyze complex macromolecular networks within the oilcake matrix [11,20]. Microbial phytases dephosphorylate phytic acid (*myo*-inositol hexakisphosphate) into lower inositol phosphates (IP_5_ through IP_1_), effectively dismantling mineral chelation centers and significantly increasing the bioavailability of divalent cations such as Zn^2+^, Fe^2+^ and Ca^2+^ [11,21].

Fermentation also drives major improvements in organoleptic quality and protein digestibility [96,110]. The metabolic activity of lactic acid bacteria converts lipid oxidation by-products (primarily volatile aldehydes like hexanal that cause grassy, beany off-notes) into pleasant organic acids and volatile esters [20]. Concurrently, limited proteolytic cleavage breaks down dense, high molecular weight globulins into low molecular weight peptides and free amino acids, enhancing peptide solubility across broader pH ranges, boosting antioxidant capacity via electron-donating peptide sequences, and neutralizing allergenic or bitter epitopes [96,109].

#### 4.1.3. Extrusion Processing

Extrusion cooking is a high-temperature, short-time (HTST) continuous processing technology that subjects moistened oilseed biomass to a combination of thermal energy, pressure (up to 3–20 MPa), and intense mechanical shear forces within a barrel equipped with rotating screws [14,104]. Depending on feed moisture content, extrusion is divided into Low Moisture Extrusion Processing (LMEP, <35–40% moisture) for expanded, porous texturized vegetable proteins (TVP) and High Moisture Extrusion Processing (HMEP, 40–70% moisture) for creating anisotropic, fibrous meat analogs [105,111,112]. Inside the extruder barrel, the combined thermomechanical forces disrupt native protein folding, dissociate hexameric edestin and helianthinin complexes, and transform the material into a continuous viscoelastic melt [113,114]. As the molten protein-fiber blend passes through a specialized long cooling die attached to the extruder exit, the decrease in temperature and laminar shear flow induce parallel realignment of the unfolded polypeptide chains along the flow streamlines [111,115]. Hydrogen bonding, hydrophobic interactions, and newly formed intermolecular disulfide cross-links stabilize this alignment, resulting in layered, anisotropic fibrous macrostructures that closely resemble mammalian muscle fibers [104,114]. Furthermore, the thermomechanical shear breaks down the rigid lignocellulosic cell walls in the oilcake, partially solubilizing insoluble dietary fibers and modifying the overall matrix hydration dynamics [43,116].

#### 4.1.4. Milling and Micronization

Mechanical milling, particularly ultrafine grinding and micronization (e.g., air classification, jet milling, vibratory ball milling), serves as an effective physical modification strategy to alter the functional behavior of oilcakes without chemical additives [33,102]. Standard coarse milling produces irregular particles with low surface reactivity, whereas ultrafine milling and micronization reduce the median particle size (d_50_) of oilcakes down to microscale dimensions (<20–50 μm) [98,102]. This intensive size reduction causes an exponential increase in the specific surface area and structural porosity of the biomass [81].

The mechanical stress generated during ultrafine milling fractures the dense cell wall architecture, exposing previously entrapped intracellular protein bodies and hydrophilic polysaccharide hydroxyl groups [33,80]. This structural disruption facilitates rapid solvent penetration and wetting kinetics, increasing the apparent water dispersion rate, swelling capacity, and initial protein extractability [33]. However, excessive micronization can generate intense localized frictional heat, causing partial mechanochemical protein denaturation and surface lipid release, which necessitates precise temperature control during industrial grinding runs [102].

### 4.2. Comparative Analysis of Thermal Processing and Downstream Technologies on Functional Properties

#### 4.2.1. Protein Denaturation and Aggregation

Thermal treatments including moist autoclaving, dry-air roasting, microwave dielectric heating, and extrusion cooking induce conformational transitions within the storage globulins that could also be true in the case of hemp and sunflower press cakes [83,106]. Differential Scanning Calorimetry (DSC) provides thermodynamic metrics that characterize the structural stability of the major storage globulins: hexameric edestin (11S legumin type globulin, ~300–350 kDa) in hemp and helianthinin (11S globulin, ~300–385 kDa) in sunflower [17,26].

In native, unheated hemp protein isolates derived from cold-pressed cakes, DSC thermograms reveal a minor endothermic peak corresponding to the 2S albumin fraction at a thermal denaturation peak temperature (T_d_ or T_p_) of 68.3 °C with a low transition enthalpy, followed by a dominant endothermic transition for native 11S edestin at T_d_ = 93.5–96.2 °C with an associated unfolding enthalpy of ΔH = 11.8–21.7 mJ/mg [69,72,117].

The magnitude of ΔH reflects the overall proportion of intact native tertiary and quaternary structures held together by internal hydrophobic interactions and intramolecular disulfide bonds (−S-S−).

When oilcakes undergo primary hot mechanical expelling (>100 °C), desolventizer-toaster treatment, or high shear extrusion, the residual **Δ**H values decrease toward zero (**Δ**H from 2.34 J/g before toasting to 0.74 J/g), confirming irreversible loss of native conformational order and the formation of non-cooperative, pre-aggregated protein networks [39,49,118].

#### 4.2.2. Thermal Denaturation Kinetics and Structural Aggregation Pathways

Thermal unfolding and subsequent aggregation of oilseed globulins follow an irreversible, non-equilibrium two-stage Lumry-Eyring kinetic mechanism, described macroscopically by pseudo first-order or second-order kinetic models:

Native N⇌k1k−1Unfolded Intermediate U→k2Irreversible Aggregate An

The temperature dependence of the thermal denaturation rate constant (*k*) follows the Arrhenius relationship:

$$k=A\exp\left(-\frac{E_{a}}{\mathrm{RT}}\right)$$

where *E_a_* is the activation energy of unfolding, *R* is the universal gas constant (8.314 J ⋅ mol^−1^ ⋅ K^−1^), *T* is absolute temperature in Kelvin, and *A* is the pre-exponential frequency factor.

For 11S edestin in neutral aqueous media (pH 7.0), the activation energy of thermal denaturation is *E_a_* = 285 − 320 kJ/mol, with a decimal reduction time for native structural loss (*D*-value) dropping rapidly from D85∘C≈28.5 min to D95∘C≈2.1 min [27,73].

Helianthinin exhibits a comparable kinetic barrier with E_a_ = 586 ± 20 KJ/mol and a denaturation temperature sensitivity (z-value) of z ≈ 10.0 °C [119,120].

During the initial reversible endothermic stage (k_1_), thermal input disrupts the non-covalent hydrogen bonding stabilizing α-helices (∼1650–1658 cm^−1^) and breaks the electrostatic salt bridges maintaining the hexameric quaternary assembly into individual trimeric and monomeric subunits [73,106]. As the temperature surpasses T_d_, the exposed non-polar hydrophobic residues (Leu, Ile, Val, Phe) and reactive free sulfhydryl groups (−SH) undergo rapid, irreversible intermolecular cross-linking (k_2_), driven by hydrophobic association and covalent thiol-disulfide (−SH/−S-S−) interchange reactions [49,83]. This transition is tracked by Fourier-Transform Infrared (FTIR) spectroscopy, which shows the disappearance of native α-helical signals and the emergence of strong Amide I intermolecular β-sheet vibrations at 1618–1628 cm^−1^ and 1685 cm^−1^ [36,49].

The thermodynamic and kinetic parameters for storing globulin are shown in Table 2.

#### 4.2.3. Changes in Water and Oil Holding Capacity

The thermodynamic shifts observed via DSC directly govern hydration and lipid binding behavior [13,83]. Thermal treatments conducted below the denaturation threshold (*T* < *T_d_*) induce minor structural expansion without extensive aggregation, increasing Water Holding Capacity (WHC) from 1.78 ± 0.07 H_2_O/g in native meal to 4.21 ± 0.24 g/g in moderately heated protein isolates [83,85]. This enhancement occurs because partial chain unfolding exposes polar amino acid backbones and hydrophilic residues (−COOH, NH_2_, −OH), enabling multi-layer hydrogen bonding with surrounding water molecules [27,85].

Similarly, the exposure of non-polar side chains increases surface hydrophobicity (H_0_); it could raise Oil Holding Capacity (OHC) in isolates treated near T_d_, as shown in the case of soybean, from 18.39 g/g at 95 °C to 12.33 g/g at 100 °C [35,77,86]. However, when thermal treatment exceeds T_d_ for extended holding times (T > 95–100 °C), severe macrogel aggregation collapses the internal capillary network and buries functional binding sites inside dense aggregates, reducing both WHC and OHC [13,83,122].

#### 4.2.4. Structural Modification of Proteins and Fibers

Extrusion processing subjects oilcake matrices to high temperatures, high pressures, and intense mechanical shear forces within an extruder barrel [14]. This combined thermomechanical action breaks native protein quaternary structures, realigning polypeptide chains along the flow axis [114]. Concurrently, high mechanical shear ruptures rigid lignocellulosic cell walls in the fiber matrix, cleaving glycosidic bonds in insoluble cellulose and hemicellulose to yield lower molecular weight soluble fiber fractions [80].

At the macromolecular level, the thermomechanical environment within the extruder barrel induces severe disruption of intramolecular non-covalent forces, principally hydrogen bonds and electrostatic interactions, causing the globular storage proteins (edestin in hemp and helianthinin in sunflower) to unfold into an amorphous, molten viscoelastic state [111,113]. As the biopolymer melt advances through the high shear mixing zones, previously buried free sulfhydryl (−SH) groups and hydrophobic residues are exposed to the matrix interface. Upon reaching the laminar flow field of the specialized cooling die, these exposed functional groups and hydrophobic domains undergo extensive intermolecular realignment [36]. Upon reaching the laminar flow field of the specialized cooling die, these exposed radicals undergo extensive intermolecular realignment, facilitating thiol disulfide exchange reactions and ordered hydrophobic cross-linking that establish continuous, three-dimensional anisotropic fibrous networks [43,104].

Simultaneously, the structural evolution of the carbohydrate fraction actively governs this protein texturization mechanism [116]. High shear and barrel temperatures depolymerize complex, tightly packed cellulose–lignin complexes and solubilize pectic polysaccharides, increasing total matrix porosity and hydration potential [79,81]. While moderate amounts of finely dispersed soluble dietary fibers intercalate into the developing protein network to enhance moisture retention and matrix elasticity, excessive levels of coarse, un-sheared insoluble fiber fragments act as steric disruptors that interrupt protein–protein junction zones [33,80]. Consequently, precise optimization of specific mechanical energy (SME) and barrel moisture is required to coordinate protein cross-linking with fiber fragmentation, ensuring both structural integrity and a fibrous, meat-like bite profile in the finished extrudate [14,105].

#### 4.2.5. Improvement of Protein Solubility and Hydration

While direct high-temperature roasting reduces protein solubility, ultrafine milling and micronization significantly enhance hydration speed and apparent solubility without altering chemical primary structures [102]. Mechanically reducing particle sizes to microscale dimensions drastically increases total specific surface area, allowing water molecules to rapidly hydrate particle surfaces and disperse insoluble protein-fiber aggregates [33,102]. Furthermore, controlled enzymatic hydrolysis or solid-state fermentation partially cleaves dense protein chains into short peptides, boosting protein solubility across wide pH ranges [96].

At the molecular level, bioprocessing via enzymatic hydrolysis and lactic acid bacteria fermentation facilitates structural breakdown by targeting specific peptide linkages within the core storage globulins (edestin and helianthinin) [11,96]. This targeted proteolysis disrupts dense quaternary aggregates and releases low molecular weight peptides with elevated surface-to-volume ratios [11]. The resulting terminal carboxyl (−COO^−^) and amino ($-NH_{3}^{+}$) functional groups increase the net ionizable charge density and hydrophilic surface area, thereby enhancing polar interactions with the surrounding aqueous microenvironment [16,45]. Consequently, the Nitrogen Solubility Index (NSI) shifts upward across both acidic and alkaline regimes, effectively minimizing the typical dip in solubility observed near the isoelectric point (pH 5.0–7.0) [45,123].

In parallel, mechanical micronization and moderate physical disruption enhance the hydration kinetics by altering the physical state of insoluble cell-wall polysaccharides and pre-aggregated proteins [33,81]. The high shear and impact forces fracture rigid lignocellulosic networks, exposing previously sequestered hydrophilic domains and polar amino acid residues (−OH, −COOH, −NH_2_) that were buried inside compact cellular structures [27,80]. This physical matrix opening increases structural capillary action and porous water-trapping capacity, which raises the Water Solubility Index (WSI) and swelling capacity without promoting the formation of insoluble disulfide-linked macrogels [102,116]. When integrated into liquid or semi-solid food formulations, these modified press cake ingredients disperse readily with reduced grittiness, providing uniform colloidal stability and superior functional behavior in beverage and emulsion systems [33,90].

#### 4.2.6. Enhancement of Emulsifying and Foaming Properties

Moderate thermal conditioning and controlled extrusion alter interfacial behavior by increasing molecular surface hydrophobicity without causing full precipitation [104]. Partially unfolded protein molecules rapidly align at oil–water and air–water interfaces, forming cohesive viscoelastic films that reduce interfacial tension [90,124]. However, over-processing leads to dense aggregation, which impairs film formation and reduces both Foaming Capacity and emulsion stability [83,92].

The interfacial activity of oilcake proteins depends on the ratio of molecular flexibility to surface hydrophobicity (*H*_0_) [90,91]. Controlled thermal treatments (80 °C below T_d_ of the 11S globulins) increase Emulsifying Activity Index (EAI) and Foaming Capacity (FC) by accelerating the diffusion rate of partially unfolded molten globule intermediates toward oil–water and air–water boundaries [77,124,125]. These flexible intermediates reorient rapidly at the interface, forming cohesive, viscoelastic protective films stabilized by intermolecular interactions that resist droplet coalescence [90].

Conversely, heating above the denaturation peak drives the formation of high molecular weight insoluble aggregates that diffuse slowly and cannot pack efficiently at interfaces [83,92,126]. This reduces EAI, broadens emulsion droplet size distributions and accelerates foam collapse and liquid drainage [13,92].

#### 4.2.7. Texture and Rheological Modification

Extrusion cooking, specifically High Moisture Extrusion Processing (HMEP) at moisture levels above 40–50%, reorganizes hemp and sunflower proteins into anisotropic, fibrous structures that closely mimic animal muscle tissue [104,105]. Thermally denatured and sheared proteins unfold, align, and cross-link through non-covalent bonds and disulfide linkages upon exiting a cooled die, forming layered fibrous networks with high tensile strength, elasticity, and firmness [111,113].

Thermally induced protein unfolding and subsequent aggregation alter the rheological profile of oilcake dispersions [18,93]. Dynamic oscillatory shear rheology reveals that heating native hemp and sunflower dispersions above their denaturation transition onset (T_onset_ > 80 °C) triggers a sharp rise in both storage modulus (*G′*) and loss modulus (*G″*), marking the sol–gel transition temperature (T_gel_) [94,95,127]. During this transition, *G′* surpasses *G″* by over an order of magnitude (tan δ = *G″*/*G′* < 0.1), indicating the development of an elastic three-dimensional gel network stabilized by hydrophobic associations and covalent disulfide cross-links [94].

Proteins that have been severely pre-denatured during commercial oil pressing cannot undergo this structured cross-linking process; instead, they exhibit elevated initial room-temperature viscosities but produce weak, fragile particulate gels upon subsequent heating [81,95].

#### 4.2.8. Influence on Sensory and Textural Characteristics

Biochemical downstream processing through solid-state or submerged fermentation (e.g., using *Lactobacillus* species or *Aspergillus* fungi) fundamentally improves sensory profiles [109]. Microorganisms degrade bitter-tasting low-molecular-weight peptides, hydrolyze antinutritional phytates, and metabolize green, grassy hexanal volatiles into pleasant ester and acid compounds [20,108]. Fermentation also lightens oilcake color by breaking down phenolic complexes, creating a more organoleptically pleasing, neutral-tasting food ingredient [110].

Extrusion texturization represents a combined thermomechanical operation where thermal denaturation kinetics interact with high shear stresses [14,111]. Inside the extruder barrel, high specific mechanical energy and thermal input (119.9 °C, exceeding T_d_) [126] melt and dissociate the globulin hexamers into a continuous, flowing viscoelastic fluid [113,114]. Simultaneously, high shear forces fragment insoluble cellulosic and hemicellulosic cell wall fractions, reducing their molecular weight and increasing soluble dietary fiber levels [80]. As the molten mass enters the laminar shear field of a long cooling die, the aligned polypeptide chains cool and cross-link via newly formed disulfide bonds, hydrogen bonds, and hydrophobic interactions along the flow streamlines [104,114].

#### 4.2.9. Particle Size Reduction and Micronization Mechanics

Milling, air classification, and ultrafine micronization (jet milling and vibratory ball milling) modify oilcake functionality through non-thermal physical comminution [33,102]. Mechanically reducing particle sizes to microscale dimensions drastically increases total specific surface area exponentially and disrupts the dense lignocellulosic matrix surrounding intracellular protein bodies [33,98]. This structural disruption facilitates rapid water penetration, increasing initial hydration speed, apparent solubility, and swelling capacity without chemically denaturing polypeptide chains [81,102].

Furthermore, micronized sunflower and hemp flours produce smoother dispersions with lower perceived grittiness and higher apparent suspension viscosity, enabling their direct incorporation into plant-based beverages, sports formulations, and baked cereal doughs [33,93].

### 4.3. Impact of Processing on Physicochemical and Techno-Functional Attributes

#### 4.3.1. Protein Quality and Digestibility

Processing technologies exert a profound influence on the nutritional quality, structural accessibility, and in vitro gastrointestinal digestibility of hemp and sunflower proteins [48,128]. In their native states, compact globular proteins like edestin and helianthinin are stabilized by dense hydrophobic cores and extensive intra-chain disulfide bonds, which physically restrict the access of endopeptidases (pepsin, trypsin, and chymotrypsin) to cleavage sites [27,115]. Controlled thermal processing and extrusion promote moderate protein unfolding, exposing scissile peptide bonds along the backbone to digestive enzymes and significantly increasing the in vitro protein digestibility (IVPD) score [106,128].

Nevertheless, the boundary between functional optimization and nutritional degradation is narrow. Severe dry heat treatment or excessive extruder barrel temperatures (>160 °C) accelerate Maillard-driven cross-linking and the formation of lysinoalanine, destroying essential lysine residues and reducing protein bioavailability [39,49]. Conversely, bioprocessing via lactic acid fermentation and enzymatic hydrolysis pre-cleaves intact globulin structures into easily absorbable oligopeptides and free amino acids, mitigating antinutritional enzyme inhibition and yielding superior nutritional uptake indices [11,96].

#### 4.3.2. Lipid Stability and Fatty Acid Composition

Although oilcakes represent the defatted residue of oilseed processing, residual lipid fractions (ranging from <2% in solvent-extracted cakes to 5–20% in cold-pressed cakes) remain rich in polyunsaturated fatty acids (PUFAs), notably linoleic (C18:2 n-6) and α-linolenic (C18:3 n-3) acids [24,51,129]. The exposure of these residual lipids to elevated processing temperatures, oxygen, and mechanical shear accelerates lipid autoxidation and hydrolytic rancidity [75,76]. Thermal treatments accelerate the homolytic cleavage of carbon–hydrogen bonds adjacent to double bonds, generating lipid alkyl radicals that react with atmospheric oxygen to form lipid hydroperoxides (primary oxidation products) and conjugated dienes [75].

Downstream processing technologies can be tailored to manage lipid degradation pathways. While extrusion at low moisture can promote the thermal cleavage of hydroperoxides into volatile secondary oxidation products (hexanal, 2,4-decadienal) that impart rancid off-flavors, high moisture extrusion and rapid cooling limit oxygen exposure, thereby preserving fatty acid saturation profiles [74,104]. Additionally, thermal processing and extrusion cook stages inactivate endogenous lipoxygenases and lipases, protecting the stabilized meal from hydrolytic free fatty acid (FFA) accumulation during subsequent storage [40,76].

#### 4.3.3. Dietary Fiber and Carbohydrates

The carbohydrate fraction of hemp and sunflower oilcakes consists predominantly of structural dietary fibers, including cellulose, hemicelluloses (xyloglucans and arabinoxylans), pectin, and complex polyphenolic lignin networks [19,78]. In crude oilcakes, insoluble dietary fiber (IDF) comprises 93.2–97.9% out of approximately 93.2% of total dietary fibers (TDF), which can cause gritty mouthfeel and disrupt protein-protein gel networks in food formulations [33,130]. Processing technologies, particularly thermomechanical extrusion and enzymatic hydrolysis, alter this balance by redistributing fiber fractions from insoluble to soluble forms (SDF) [81,116].

During extrusion cooking, high mechanical shear forces cleave covalent glycosidic bonds in complex hemicellulose and pectin polymers, breaking high molecular weight polysaccharides into smaller, water-soluble oligosaccharide fragments [80,81]. This increase in the SDF:IDF ratio improves physiological prebiotic functionality and increases the water binding, viscous swelling, and bile acid retention capacities of the ingredient [79,116]. Similarly, solid-state fermentation utilizing cellulolytic fungal strains partially degrades rigid cellulosic matrices, increasing total soluble dietary fiber yields and improving ingredient mouthfeel in formulated foods [109].

#### 4.3.4. Bioactive Compounds and Antioxidant Activity

The retention, release, and transformation of bioactive secondary metabolites such as cannabisin lignanamides in hemp and chlorogenic acid isomers in sunflower are directly dictated by the processing pathway [35,38]. In raw seed cakes, a significant portion of phenolic compounds is chemically esterified or entrapped within insoluble cell wall polysaccharides, which restricts their extractability and immediate bioaccessibility [34,60]. Moderate thermal processing and extrusion break these ester bonds, liberating bound phenolics into free forms that can increase measurable Total Phenolic Content (TPC) and ferric reducing antioxidant power (FRAP) [41,42].

However, excessive thermal exposure or extrusion at high barrel temperatures (>140^∘^C) triggers the thermal decomposition and decarboxylation of sensitive phenolic acids, reducing radical scavenging capacities (DPPH and ABTS assays) [41,42].

Bioprocessing via fermentation avoids this thermal degradation; microbial β-glucosidases hydrolyze phenolic glycosides into aglycones with higher antioxidant potential, while microbial proteases release antioxidant peptides rich in histidine, tyrosine, and tryptophan, enhancing the overall oxidative stability of the food matrix [11,108].

#### 4.3.5. Water Holding Capacity (WHC)

Water Holding Capacity (WHC), defined as the quantity of water that a protein or fiber matrix can physically imbibe and retain against gravitational or centrifugal force, is heavily modulated by structural transformations during processing [13,83]. In untreated, native oilcakes, WHC is relatively low because compact globular conformations bury hydrophilic amino acid side chains (−COOH, −OH, −NH_2_) within internal hydrophobic cores [27,85]. Controlled thermal processing and extrusion unfold these globulins, exposing polar peptide backbones and hydrophilic residues, which facilitates direct hydrogen bonding with water molecules and increases overall WHC [14,83].

Simultaneously, structural changes in the dietary fiber network contribute substantially to water retention [81]. Mechanical shear during extrusion and micronization expands matrix porosity, creating structural microcavities that trap water through capillary action [33,116]. Ingredients treated under optimized high moisture extrusion conditions exhibit high WHC values, making them suitable for enhancing juiciness in plant-based meat analogs and reducing moisture loss during baking in cereal applications [93,105].

#### 4.3.6. Oil Holding Capacity (OHC)

Oil Holding Capacity (OHC) reflects the ability of non-polar amino acid side chains and structural micropores to bind and immobilize liquid lipids, a property essential for flavor retention and fat binding in structured foods [13,86]. The exposure of hydrophobic residues (Leu, Ile, Val, Phe) upon thermal and mechanical unfolding increases the surface lipophilicity of hemp and sunflower proteins, driving higher OHC values compared to native, unheated meals [69,83].

Extrusion and micronization further amplify OHC by creating expanded, porous capillary networks within the fragmented biomass [14,102]. Fully defatted meals generated via supercritical CO_2_ or solvent extraction exhibit higher OHC values than cold-pressed cakes because their internal pores are unoccupied by residual oil, leaving them free to absorb and retain lipids during food formulation [35,60]. This high oil-binding performance makes processed oilcake isolates suitable for emulsified meat products, vegan sausages, and baked confectionery systems [88,89].

#### 4.3.7. Protein Solubility

Protein solubility serves as a primary techno-functional indicator, as it dictates the suitability of an ingredient for use in liquid, colloidal, and emulsified food systems [16,45]. Processing technologies alter the balance between repulsive electrostatic forces and attractive hydrophobic interactions on the protein surface [84,131]. High-temperature dry roasting and harsh chemical desolventization reduce protein solubility across all pH values by promoting the formation of large, insoluble aggregates stabilized by intermolecular disulfide cross-links and extensive hydrophobic stacking [69,72].

Conversely, controlled bioprocessing and physical technologies can restore or enhance protein solubility [96,102]. Limited enzymatic hydrolysis and lactic acid fermentation cleave high molecular weight globulins into shorter, charged peptides, raising the surface-to-volume ratio and increasing the number of ionizable terminal carboxyl (−COO^−^) and amino ($-{\mathrm{NH}}_{3}^{+}$) groups [11,96]. These structural modifications enhance hydration interactions and shift the isoelectric point (pI), leading to high nitrogen solubility index (NSI) values across both acidic and alkaline conditions [45,84].

#### 4.3.8. Foaming Properties

Foaming Capacity (FC) and Foam Stability (FS) depend on the ability of soluble protein molecules to migrate rapidly to the air–water interface, unfold, reduce interfacial tension, and form a continuous, cohesive viscoelastic film that traps gas bubbles [90,92]. Native edestin and helianthinin exhibit moderate foaming properties due to their rigid quaternary structures, which can slow down interfacial migration and alignment [17,115]. Processing methods that introduce controlled molecular flexibility, such as mild thermal conditioning, partial enzymatic hydrolysis, or high-pressure homogenization, significantly increase FC by facilitating rapid interfacial adsorption [73,91].

However, excessive thermal processing that induces large-scale insoluble aggregation severely impairs foaming functionality [83]. Large aggregates migrate slowly toward the interface and lack the molecular flexibility required to establish strong, cohesive interfacial films, resulting in rapid bubble coalescence and liquid drainage [13,92]. Additionally, residual lipids in cold-pressed oilcakes act as antifoaming agents by competitively adsorbing at the air–water interface without forming elastic films; consequently, highly defatted oilcake isolates typically exhibit superior foaming characteristics [51,85].

#### 4.3.9. Gelation Properties

Heat-induced gelation involves a multi-step thermodynamic process in which native proteins undergo thermal unfolding, followed by ordered intermolecular aggregation and cross-linking into a continuous three-dimensional viscoelastic gel network capable of entrapping water and solutes [94,95]. The least gelation concentration (LGC), a critical measure of gelling efficiency where a lower percentage indicates superior performance, is directly governed by the processing history of the oilcake [13,86]. Processing techniques that preserve native globulin structures (e.g., supercritical CO_2_ extraction, mild cold pressing) maintain low LGC values (10–12.5% *w*/*v*), forming firm, self-supporting elastic gels upon thermal curing [69,94].

When proteins are severely pre-denatured through harsh thermal processing or high shear dry extrusion, their capacity to form ordered three-dimensional gel networks is compromised [83]. Pre-aggregated proteins undergo random, particulate aggregation rather than ordered cross-linking, producing weak, fragile gels with high turbidity and poor syneresis resistance [94,95]. To address this, non-thermal processing aids such as transglutaminase cross-linking or blending with hydrocolloids are often employed to facilitate gelation in processed oilcake ingredients [95,132].

#### 4.3.10. Rheological and Textural Properties

The rheological behavior of oilcake-derived suspensions, pastes, and gels is characterized by shear viscosity, yield stress, storage modulus (*G′*), and loss modulus (*G″*) is dictated by the structural configuration of both protein and fiber fractions [18,93]. Native oilcake dispersions typically exhibit non-Newtonian, shear-thinning (pseudoplastic) behavior, where viscosity decreases with increasing shear rate due to the progressive alignment of asymmetric particles along the shear field [94]. Thermal processing and extrusion increase both *G′* and *G″* by promoting macromolecular entanglement and intermolecular disulfide bridging, shifting the system from a viscous fluid state toward an elastic solid-like gel state (*G′* > *G″*) [95].

Particle size reduction via micronization also plays a significant role in rheological modification [33,102]. Micronized sunflower and hemp press cakes produce suspensions with higher apparent viscosities and yield stresses at equivalent solids contents compared to coarsely milled counterparts, as the increased hydrodynamic surface area enhances particle–particle frictional interactions [33,98]. When incorporated into food systems such as bread dough or plant-based meat analogs, these modified rheological properties strengthen dough development and increase the tensile firmness and chewiness of the finished matrix [93,105].

### 4.4. Factors Affecting Functional Properties

The precise control of processing parameters, including temperature, pressure, moisture content, mechanical shear rate, residence time, and pH, dictates the extent of physical and chemical transformations in oilcakes [14,115]. In extrusion, elevated barrel temperatures combined with low moisture levels amplify mechanical shear, driving extensive starch degradation, severe protein denaturation, and Maillard browning [41,80]. Conversely, high moisture extrusion processing (HMEP) utilizes moisture levels above 40% to cushion mechanical shear, enabling controlled protein unfolding and alignment into anisotropic fibrous textures without thermal degradation [111,114]. Similarly, during thermal treatments, the presence of moist heat (e.g., autoclaving) promotes structural flexibility and enzymatic inactivation at lower temperatures compared to dry heat roasting [107].

The raw material composition, specifically the ratio of globulins to albumins, residual fat concentration, dietary fiber distribution, and polyphenol levels, determines the technological responsiveness of the oilcake [113,131]. High residual lipid concentrations could act as internal lubricants during mechanical milling and extrusion, such as proven in soy with residual lipid contents of 9–17%, reducing shear stress and inhibiting the cross-linking required to form fibrous protein networks [111,133]. High insoluble fiber levels (cellulose, lignin from unhulled seeds) disrupt protein–protein interactions, acting as physical inclusions that weaken gel structures and reduce emulsification efficiency [19,33]. In sunflower cakes, chlorogenic acid concentrations drive the extent of protein–polyphenol cross-linking; high CGA content under alkaline processing conditions causes covalent cross-linking that drastically lowers protein solubility and alters gel network strength [12,44].

The functional stability of processed oilcakes during post-processing storage depends heavily on environmental parameters, including ambient temperature, relative humidity (RH), water activity (a_w_), oxygen exposure, and packaging barrier properties [100,101]. High ambient storage temperatures and elevated relative humidity accelerate protein aging and hydrophobic aggregation, leading to gradual losses in protein solubility, WHC, and Foaming Capacity over time [100]. Additionally, lipid-rich cold-pressed oilcakes stored under oxygen-rich conditions undergo rapid auto-oxidation, generating free radicals and reactive hydroperoxides that cross-link adjacent proteins, causing off-flavors, dark discoloration, and impaired functional performance [74,75].

## 5. Future Perspectives and Industrial Applications

### 5.1. Functional Ingredient Development

The upcycling of hemp and sunflower oilcakes into high-purity protein isolates (95.5% protein for dehulled meal and 90.3–90.8% cold-pressed whole meal dry basis) [134,135], functional concentrates, and specialized dietary fiber fractions represent a cornerstone for sustainable food formulations [9,10]. Advanced fractionation routes such as membrane ultrafiltration–diafiltration, pH shifting combined with high-intensity ultrasonication, and two-phase natural deep eutectic solvent (NADES) systems enable the gentle separation of native edestin and helianthinin without inducing thermal denaturation [25,77,136]. These purified fractions exhibit defined solubility, foaming, and emulsification indices, making them capable of substituting conventional animal proteins (e.g., egg white, sodium caseinate) and commercial soy ingredients in food emulsions, structure-forming hydrogels, and oleogel matrices [90,137,138].

Simultaneously, the co-extraction and purification of dietary fiber fractions provide clean label stabilizers, fat mimetics, and prebiotic bulking agents [78]. Enzymatic adaptation allows the insoluble-to-soluble fiber ratio to be engineered for target applications, enhancing viscosity in beverage systems and strengthening water retention in complex dough formulations [79,81]. By adapting these extraction conditions, manufacturers can develop multifunctional ingredient streams that align with consumer demand for non-GMO, allergen-friendly, and nutrient-dense plant proteins [16,103].

### 5.2. Innovative Processing Technologies

Emerging non-thermal processing interventions and advanced thermal systems provide precise tools for structural modification while preserving heat-sensitive bioactive compounds [1,14]. Technologies including High Hydrostatic Pressure (HHP; up to 1000 MPa), Pulsed Electric Fields (PEF), cold atmospheric plasma, and hydrodynamic cavitation generate controlled conformational changes in seed globulins [73,139]. These non-thermal treatments selectively disrupt quaternary protein assemblies, cleave non-covalent hydrophobic and electrostatic bonds, and reduce particulate agglomeration without driving irreversible thermal precipitation [73,136].

**Figure 3:**
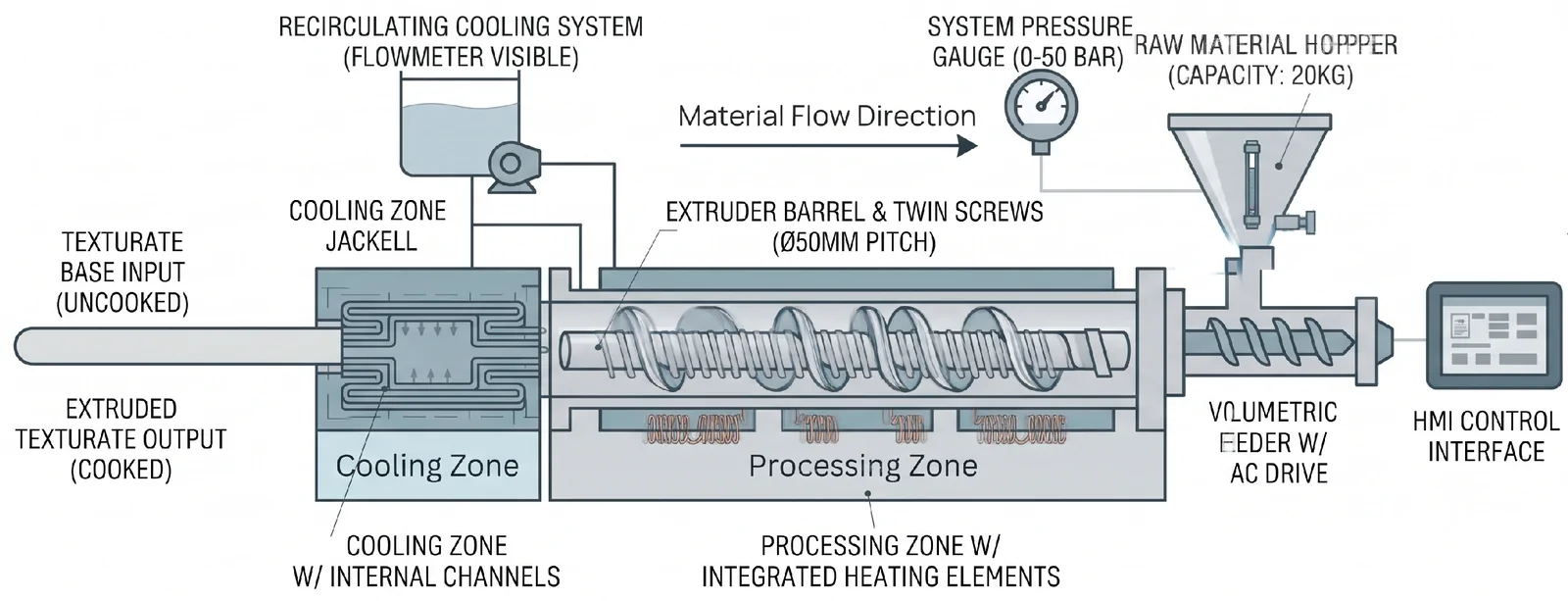
Schematic illustration of the transformation of upcycled oilcake proteins into structured plant-based food systems reinterpreted from Fu et al. [144].

To optimize these processing operations, computer-aided food engineering (CAFE) frameworks, multiphysics Finite Element Method (FEM) simulations, and artificial intelligence-driven generative networks are increasingly utilized [140,141,142]. Mechanistic numerical models couple simultaneous mass, momentum, and heat transport equations with protein denaturation kinetics, predicting product deformation, temperature gradients, and shear stress fields inside industrial extruder barrels (Figure 3) and cooling dies [140,143]. This computational approach reduces trial-and-error development, accelerates industrial scale-up, and allows functional properties to be customized for specific target food matrices [14,141].

### 5.3. Application in Bakery Products

Integrating micronized hemp and sunflower press cakes into conventional wheat-based and gluten-free cereal matrices significantly enhances the micronutrient and macronutrient profile of baked goods [89,145]. Replacing refined wheat flour with oilcake meal at substitution levels of 10–20% creates a complementary amino acid profile: the oilcake fraction supplies essential sulfur-containing amino acids (methionine and cysteine) and arginine, while supplementing the total protein pool to help balance the natural lysine and threonine deficiencies characteristic of refined cereal gluten [89,93,146].

Technofunctionally, the incorporation of oilseed press cakes alters dough mixing properties and gas retention dynamics [145]. The high water-binding capacity of the residual dietary fibers and unfolded storage proteins increases farinograph water absorption, extends dough development time, and increases dough stability [93]. In gluten-free bread formulations, the swelling properties and gel-forming capacities of edestin and sunflower globulins help build a coherent starch–protein matrix, improving crumb elasticity, reducing post-baking moisture loss, and delaying starch retrogradation during storage [89,145].

### 5.4. Application in Plant-Based Protein Foods

Hemp and sunflower oilcakes serve as sustainable raw materials for developing next-generation plant-based meat analogs, dairy alternatives, and high-protein beverages for sports [33,147]. Utilizing High Moisture Extrusion Processing (HMEP) at moisture levels of >40%, protein concentrates derived from these press cakes undergo thermomechanical plasticization and laminar alignment in cooling dies, forming fibrous, anisotropic structures with high tensile strength and a bite profile comparable to cooked muscle tissue [104,105,112]. Blending hemp or sunflower proteins with complementary legume isolates (e.g., pea or soy) optimizes melt rheology and enhances texturization indices [113,128].

In dairy alternative emulsions and beverage formulations, micronized and enzymatically modified oilseed proteins function as effective natural emulsifiers [90,124]. Submicronized dehulled sunflower press cake dispersions form stable oil-in-water emulsions with narrow droplet size distributions, preventing gravitational phase separation and creaming during ambient storage [33]. Furthermore, partial fermentation of sunflower and hemp protein slurries hydrolyzes bitter peptide fragments, generating smooth, nutrient-dense plant-based milk and yogurt bases with high protein digestibility [96,110].

### 5.5. Challenges and Future Perspectives

Despite their high technological potential, the large-scale industrial utilization of hemp and sunflower oilcakes faces several operational bottlenecks. Commercial challenges include:

- Developing cost-effective, scalable, and eco-friendly technologies to remove chlorogenic acid from sunflower meal without compromising protein yield or inducing off-colors [18,44].
- Mitigating persistent sensory limitations, such as bitter astringent off-flavors and green/dark colors, through optimized fermentation or clean extraction pathways [20,110].
- Navigating complex regulatory landscapes regarding novel food approvals for industrial hemp derivatives to ensure complete absence of psychoactive cannabinoid residues [7].
- Securing consumer acceptance and market adoption of upcycled agro-industrial by-products through transparent labeling and evidence-based communication of environmental benefits [148].

Addressing these interdisciplinary challenges through coordinated research in food chemistry, process engineering, and consumer science will be essential to fully capitalize on the potential of oilcakes within sustainable circular food production systems [103,149].

### 5.6. Environmental and Techno-Economic Feasibility

Techno-Economic Analysis demonstrates that the industrial upcycling of oilcakes using advanced fractionation methods (such as membrane ultrafiltration–diafiltration and supercritical fluid extraction) becomes commercially viable only when specific recovery thresholds and integrated processing models, as established in recent literature scenarios, are met [3,6,150]. Standalone protein extraction is heavily constrained by high capital expenditure (CAPEX) for membrane units and high operating costs (OPEX) linked to energy-intensive drying and enzyme inputs [66,67,150]. However, economic feasibility is established through two main operational strategies: First, facility co-location alongside existing oilseed pressing mills eliminates biomass haulage logistics, prevents lipid oxidation and protein deterioration during storage/transit, and integrates into existing utility networks (process water, steam, and electrical infrastructure) [4,5]. Second, commercial viability relies on a cascading biorefinery approach rather than single-component recovery [3,60]. By operating at modeled techno-economic benchmark assumptions reported in the literature (targeting scenario-specific protein extraction yields reaching up to 84.6% to produce high-value food isolates up to 95.5% purity on a dry basis) [135] while simultaneously fractionating value-added co-products, specifically bioactive phenolic extracts (such as chlorogenic acid from sunflower or cannabisin lignanamides from hemp) and dietary fiber functional ingredients, the revenue streams are diversified [3,6,60,78]. Membrane processes further reduce lifecycle OPEX by replacing energy-demanding thermal concentration cycles with non-thermal pressure-driven filtration, offsetting high upfront equipment investments and ensuring practical scalability [25,150].

### 5.7. Life Cycle Assessment (LCA) and Environmental Indicators

Life Cycle Assessment (LCA) demonstrates that valorizing hemp and sunflower oilcakes into food-grade protein isolates offers significant environmental advantages over both animal-derived proteins and conventional commercial soy protein isolate (SPI) [3,149]. From a circular bioeconomy perspective, because the agricultural cultivation burden (land use, synthetic fertilizer inputs, and irrigation) is primarily allocated to the primary vegetable oil fraction, the environmental footprint attributed to the residual cake by-product is markedly reduced [5,151].

Table 3 provides a detailed comparative overview of Life Cycle Assessment (LCA) benchmarks, highlighting the competitive sustainability profile of upcycled oilseed proteins against conventional alternatives. The recovery of proteins from oilcakes via green processing routes (such as supercritical CO_2_ defatting combined with ultrafiltration) could generate lower greenhouse gas emissions, as shown in the case of soybean, compared to the solvent-intensive, energy-demanding hexane extraction and acid precipitation cycles standard in commercial SPI manufacturing [55,62,152].

Life cycle assessment (LCA) studies have highlighted substantial differences in the environmental impacts of plant-derived and dairy protein products, depending on the raw material, processing technology, functional unit, and system boundaries. Meffo Kemda et al. [153] assessed the environmental impacts of hemp cultivation and seed-based food products, while Braun et al. [154] reported a carbon footprint of 2.4 kg CO_2_-eq/kg protein for commercial soy protein isolate. Heusala et al. [155] investigated the carbon footprint and land use associated with oat and faba bean protein concentrates, providing further evidence of the environmental implications of alternative protein production. Sharma et al. [156] compiled comparative estimates of greenhouse gas emissions, water use, and land requirements for plant-derived protein products and whey protein isolate, as summarized in Table 3. Berardy et al. [157] investigated the life cycle impacts of soy protein isolate, highlighting the influence of co-product allocation on environmental performance. The interpretation of these findings also requires consideration of cradle-to-gate system boundaries, as described in [158], particularly when comparing ingredients produced through different processing pathways.

**Table 3:** Comparative Life Cycle Assessment (LCA) Indicators for Upcycled Hemp and Sunflower Protein Isolates versus Conventional Plant and Dairy Benchmarks.

Environmental Impact Indicator	Hemp-Derived Products	Sunflower-Derived Products	Soy Protein Isolate (SPI)	Whey Protein Isolate (WPI)
**Carbon footprint (kg CO_2_-eq/kg protein)**	1.5–2 (hemp protein powder) [156]	-	2.4 [154]	9–11 [156]
**Water use (L/kg protein)**	1800–2800 (hemp protein powder) [156]	-	2500–4000 [156]	5000–7500 [156]
**Land use (m^2^/kg protein)**	12–16 (hemp protein powder) [156]	-	16–20 [156]	60–75 [156]
**Additional product-specific LCA findings**	Hemp flour: 33 kg CO_2_-eq/kg flour; 5.6 m^3^ water-eq/kg flour [153]	Sunflower oil: 0.4 m^3^ water-eq/kg oil [153]	-	-

While life cycle assessment (LCA) data illustrate general environmental trends, direct comparisons across studies remain challenging due to methodological differences in system boundaries, allocation procedures, functional units, and protein-purity baselines among the cited literature. Bearing these variations in mind, Table 3 provides indicative literature ranges comparing upcycled oilseed proteins with conventional alternatives.

Furthermore, hemp crops require minimal pesticide applications and contribute to soil carbon sequestration, while sunflower provides high per-hectare biomass yields, resulting in low overall eutrophication and acidification indices across their processing lifecycles [16,151].

### 5.8. Techno-Economic Analysis (TEA) and Industrial Scalability

Techno-Economic Analysis (TEA) indicates that commercializing oilcake protein recovery is economically feasible, provided high protein extraction yields up to 84.6% and efficient solvent/water recycling loops are established [3,6,135]. Capital expenditure (CAPEX) is largely dictated by the separation and drying equipment, while membrane filtration systems (UF/DF) require substantial initial capital outlays; their operating expenditures (OPEX) are significantly lower than conventional thermal evaporation trains due to reduced thermal energy demands [25,150].

Operating cost structures are favorable when valorization facilities are co-located alongside existing oilseed crushing plants [4]. Co-location eliminates biomass transport logistics, reduces feedstock storage degradation, and allows shared utility networks (steam, cooling water, electrical grids) [5]. However, the economic balance remains sensitive to enzyme costs during enzyme-assisted aqueous extraction (EAAE) and electricity costs associated with high-pressure supercritical CO_2_ compression trains [66,67]. Industrial viability is maximized when a whole biomass cascading biorefinery approach is adopted, simultaneously isolating high-purity protein isolates, bioactive phenolic extracts, and prebiotic insoluble fiber co-products to diversify commercial revenue streams [3,60].

### 5.9. Sensory Masking Strategies and Formulation Science

The commercial adoption of hemp and sunflower press cake ingredients in consumer food formulations is often limited by sensory challenges, notably bitter and astringent taste profiles, earthy or beany aromatic notes, and green or dark brown discolored appearances [18,77]. Addressing these organoleptic hurdles requires targeted biochemical, physical, and formulation approaches.

**Figure 4:**
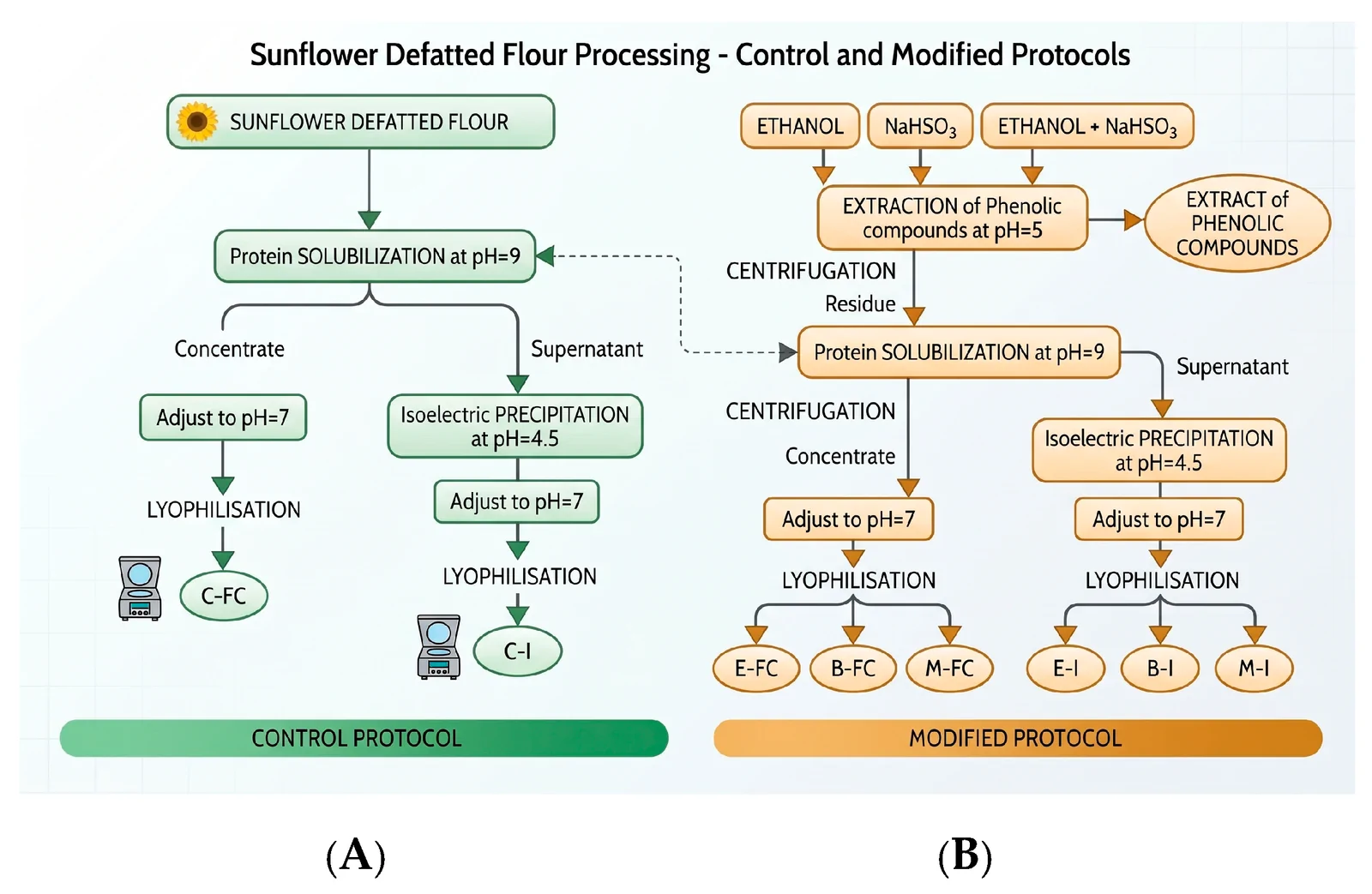
Sunflower Protein Phenolic Extraction Flowchart, reinterpreted from Alexandriano et al. [159]. (**A**) Conventional alkaline extraction–isoelectric precipitation producing standard concentrate (C-FC) and isolate (C-I). (**B**) Multi-solvent pre-treatment routes designed to strip chlorogenic acid prior to protein solubilization using ethanol (E), sodium bisulfite (NaHSO_3_; B), or combined ethanol + NaHSO_3_ (M), producing corresponding polyphenol-depleted concentrates (E-FC, B-FC, M-FC) and high-purity, light-colored protein isolates (E-I, B-I, M-I).

Figure 4 illustrates the process flow for polyphenol removal during sunflower protein extraction.

### 5.10. Chlorogenic Acid Greening Prevention in Sunflower Formulations

**Figure 5:**
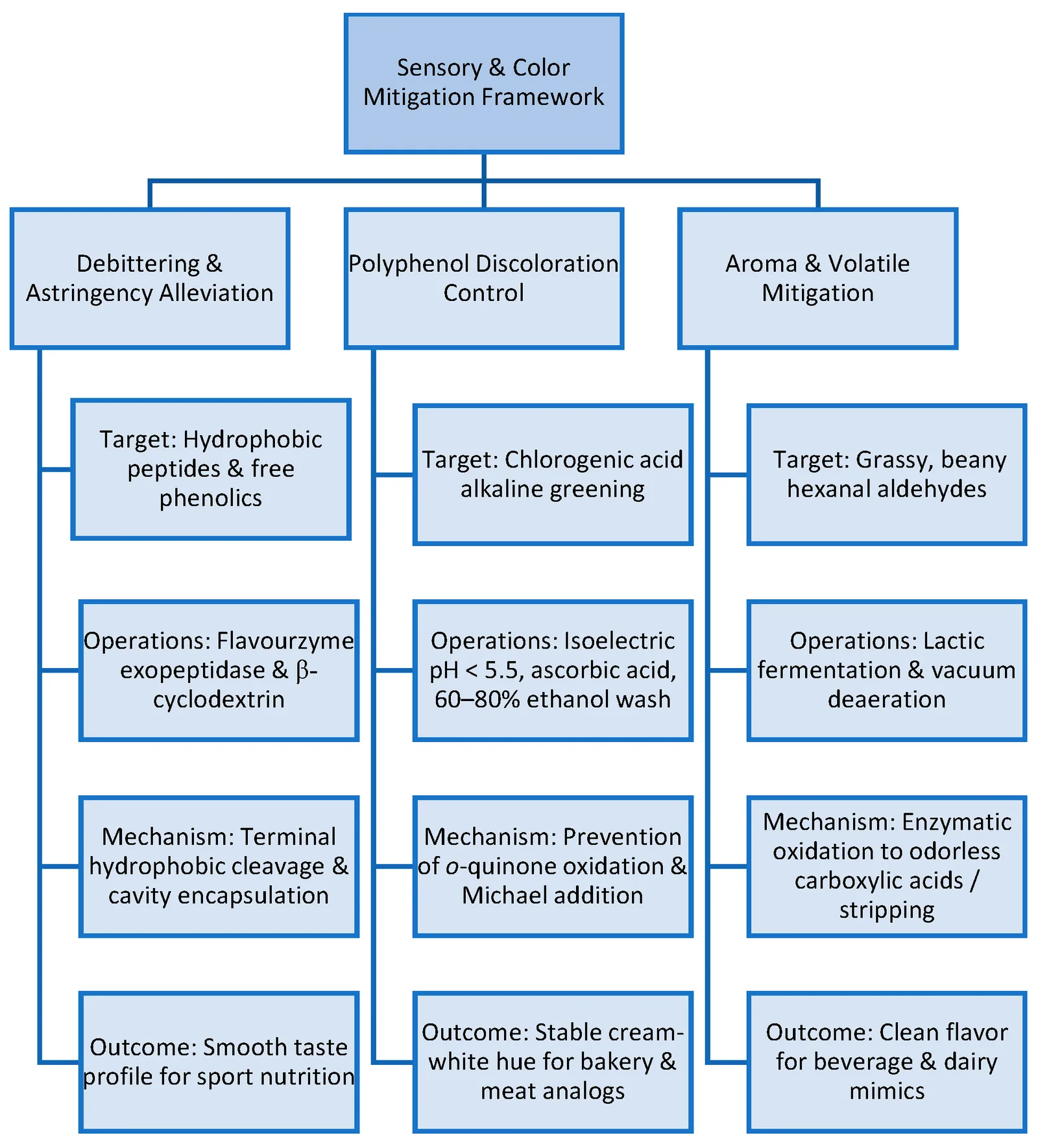
Process Architecture: Sensory and Color Mitigation [11,18,20].

The oxidation of chlorogenic acid (CGA) into green trihydroxyindole complexes under alkaline food processing conditions presents a major aesthetic defect in sunflower-supplemented bakery and meat products [18,160]. Industrial mitigation strategies focus on:

- pH Regulation: Maintaining processing environments strictly below using food-grade organic acidulants (citric, malic, or tartaric acid) prevents the deprotonation of phenolic hydroxyls, halting the autoxidation cascade into *o*-quinones [29,45].
- Reducing Agents: Formulating with natural reducing agents, such as ascorbic acid (vitamin C) or sodium metabisulfite, reduces quinone intermediates back into colorless catechols before they can covalently cross-link with the *ε*-amino groups of protein lysine residues [38,44].
- Pre-Extraction Polyphenol Washing: Washing raw sunflower meal with food-grade aqueous ethanol (80% *v*/*v* at 25 °C) or using cross-flow ultrafiltration prior to alkaline solubilization extracts 95% of native chlorogenic acid, yielding clean, cream colored protein isolates with high thermal color stability [161]; as illustrated in Figure 5, strategic interventions target specific color and sensory defects through tailored processing operations and molecular mechanisms. [2,18].

### 5.11. Volatile Off-Flavor Cleavage and Masking

The distinctive “beany,” “earthy,” and grassy off-flavors of hemp ingredients stem primarily from secondary lipid oxidation aldehydes (e.g., hexanal, (E)-2-nonenal) and alkylpyrazines [20,77].

The distinctive grassy and beany off-flavors that often limit the sensory acceptance of oilseed proteins can be mitigated through targeted biological and physical de-aromatization strategies. One effective approach is fermentation degradation, which involves inoculating oilcake slurries with selected lactic acid bacteria, such as *Lactiplantibacillus plantarum* and *Lactobacillus fermentum* [20,50]. This biological intervention induces bacterial aldehyde dehydrogenases that actively metabolize undesirable volatile aldehydes into non-volatile, odorless carboxylic acids or pleasant fruity esters, thereby significantly improving the aromatic profile of the protein ingredient [20].

Complementing these biochemical methods, physical de-aromatization techniques are employed to mechanically remove non-polar volatile aroma compounds from the protein matrix. Applying high-vacuum flash deaeration during aqueous extraction unit operations effectively volatilizes and strips these off-flavors from the slurry [63]. Furthermore, incorporating downstream supercritical CO_2_ strip cycles provides an exhaustive de-aromatization route, utilizing the unique solvent properties of supercritical fluids to extract hydrophobic odorants, ultimately yielding deodorized protein flours with neutral sensory characteristics suitable for a wide range of food applications [77].

### 5.12. Bitterness and Astringency Masking

Low molecular weight hydrophobic peptides and free polyphenols contribute to bitter and astringent taste profiles in processed oilseed meals [11,34].

Targeted enzymatic and physical masking strategies are deployed to mitigate the bitter taste profiles often associated with upcycled plant proteins. One effective approach involves enzymatic exopeptidase cleavage, where protein slurries are treated with specific fungal exopeptidases, such as Flavourzyme [11,162]. These enzymes are designed to selectively cleave terminal hydrophobic amino acid residues, including leucine, phenylalanine, and valine, from protein chains. This selective action allows manufacturers to dismantle the specific peptide sequences responsible for activating bitter taste receptors, effectively improving the ingredient’s flavor profile without causing excessive or uncontrolled protein degradation [11].

Furthermore, molecular encapsulation and masking techniques are utilized to physically shield undesirable taste compounds from human gustatory receptors. This can be achieved by incorporating food-grade guest–host inclusion complexes, such as β-cyclodextrin, which possess hydrophobic inner cavities that can trap bitter molecules [44,132]. Alternatively, complex coacervation strategies utilize anionic hydrocolloids, including gum Arabic or sodium alginate, to establish protective biopolymer networks around the target compounds. Through these physical interventions, both bitter polyphenols and astringent peptide sequences are effectively encapsulated, significantly reducing their bioavailability to taste receptors on the tongue and yielding ingredients suitable for sensitive applications like neutral-flavored sports nutrition isolates [44].

## 6. Conclusions

Hemp and sunflower oilcakes represent valuable, underutilized by-products capable of playing a central role in the transition toward sustainable, plant-based food systems. Raw press cakes are rich in high-quality storage proteins (edestin and helianthinin), essential fatty acids, dietary fiber, minerals, and bioactive phenolics. However, their physical structure, nutritional bioavailability, and techno-functional performance are directly shaped by primary oil extraction methods and downstream processing technologies.

While mechanical cold pressing and supercritical CO_2_ extraction preserve native protein structures, high-temperature mechanical expelling and conventional solvent desolventization induce severe thermal denaturation, compromising protein solubility and interfacial activity. Downstream processing techniques including extrusion cooking, fermentation, enzymatic hydrolysis, and ultrafine micronization offer powerful tools to alter these structures. These processes facilitate protein unfolding, fiber solubilization, off-flavor mitigation, and antinutrient reduction, transforming crude agricultural residues into functional ingredients tailored for bakery products, meat analogs, and novel food colloids.

Future advances rely on optimizing eco-friendly extraction techniques, scaling computational process modeling, and refining polyphenol removal strategies. Overcoming these technological and sensory hurdles will enable the food industry to fully upcycle hemp and sunflower oilcakes, advancing circular bioeconomy goals and supporting global food security.

Importantly, the multi-tiered technological framework reviewed here, encompassing cold or green-solvent extraction, ultrafine micronization, bioprocessing via microbial fermentation, and high-moisture extrusion texturization, provides a modular template directly applicable to other agro-industrial oilcakes. Comparable underutilized residues, such as rapeseed/canola, flaxseed, pumpkin seed, and sesame cakes, share analogous structural limitations, including persistent seed-coat lignocellulose, native antinutritional complexes (glucosinolates, cyanogenic glycosides, phytates), and bitter phenolic interactions. Adapting these thermomechanical and biological unit operations across diverse oilseed matrices establishes a scalable engineering pathway for circular bioeconomy platforms, accelerating the global transition toward sustainable, resilient, and allergen-friendly plant-based food systems.

## Figures and Tables

**Table 1:** Impact of Primary Extraction Methods on Nutritional Composition, Protein Functionality and Sustainability Profiles of Oilseed Meals.

Extraction Method	Nutritional Advantages	Nutritional Disadvantages	Functional Performance	Economic and Environmental Considerations
**Cold Pressing**	Preserves native proteins, heat-sensitive vitamins, and active bioactives; chemical-free [51].	High residual oil (8–15%) increases susceptibility to rancidity; lower protein concentration [51].	Excellent solubility, foaming, and gelation due to native state; moderate initial OHC [91].	Low capital expenditure; highly sustainable, low energy use, zero chemical waste [52].
**Hot Pressing**	High oil recovery yield; releases some cell-wall-bound phenolics [40].	Severe protein denaturation; loss of heat-sensitive vitamins; lipid oxidation [40].	Poor solubility, foaming, and emulsification; increased WHC due to open aggregate structure [83].	Moderate cost; energy-intensive thermal conditioning steps [52].
**Solvent Extraction**	Complete defatting (<1–2% residual fat); maximum protein concentration in meal [55].	Desolventization heat denatures proteins; potential organic solvent residue risks [56].	Variable; high OHC and WHC, but drastically reduced protein solubility and interfacial activity [15].	High capital cost; environmental and safety risks (flammable n-hexane emissions) [55].
**Supercritical CO_2_**	Solvent-free, high-purity meal; preserves complete bioactive and amino acid profiles [62].	High pressure requirements; limited efficiency for highly polar compounds without co-solvents [63].	Superior protein solubility, emulsification, foaming, and gelation capacity [65].	High initial capital equipment investment; environmentally green, recyclable CO_2_ [66].
**Enzyme-Assisted Aqueous**	Chemical-free; generates hydrolysates with enhanced bioactivity and fiber solubility [67].	Requires downstream drying; potential losses of water-soluble nutrients in wastewater [68].	High protein solubility across a wide pH range; excellent emulsification; modified rheology [67].	High enzyme costs and longer processing times; low environmental impact, aqueous effluent management required [67].

**Table 2:** Thermodynamic and Kinetic Parameters of Storage Globulins.

Protein Fraction and Source	Td (°C)	ΔH (J/g)	Primary Conformational Shift (FTIR)
**Hemp 2S Albumin (*C. sativa*)**	92.0–95.0 [117]	5–11 [69]	Minor loop unfolding; rapid thermal aggregation [85]
**Hemp 11S Edestin (*C. sativa*)**	91.9 ± 0.45 [27]	7.3 ± 0.77 [27]	α-helix (1650–1660 cm^−1^) → Intermolecular β-sheet (1612–1640 cm^−1^) [85]
**Sunflower 2S Albumin (*H. annuus*)**	99.7–101.9 [121]	14.2–15.2 [121]	Disruption of compact disulfide-stabilized loops [15]
**Sunflower 11S Helianthinin (*H. annuus*)**	90.1 ± 0.7 [121]	-	Hexamer dissociation → Covalent −S-S− cross-linked aggregate [15]

## Data Availability

No new data were created or analyzed in this study. Data sharing is not applicable to this article.
